# Plantaricin NC8 αβ exerts potent antimicrobial activity against *Staphylococcus* spp. and enhances the effects of antibiotics

**DOI:** 10.1038/s41598-020-60570-w

**Published:** 2020-02-27

**Authors:** Torbjörn Bengtsson, Robert Selegård, Amani Musa, Kjell Hultenby, Johanna Utterström, Petter Sivlér, Mårten Skog, Fariba Nayeri, Bengt Hellmark, Bo Söderquist, Daniel Aili, Hazem Khalaf

**Affiliations:** 1 Cardiovascular Research Centre, School of Medical Sciences, Örebro University, Örebro, SE-70362 Sweden; 2 Division of Molecular Physics, Department of Physics, Chemistry and Biology (IFM), Linköping University, Linköping, SE-58183 Sweden; 3 Department of Laboratory Medicine, Division of Clinical Research Centre, Karolinska Institutet, Stockholm, SE-14186 Sweden; 4 S2Medical AB, Linköping, SE-58273 Sweden; 5 PEAS Research Institute, Department of Infection Control, Linköping, SE-58273 Sweden; 6 Department of Clinical Microbiology, Örebro University Hospital, Örebro, SE-70185 Sweden

**Keywords:** Antibiotics, Pathogens, Peptides

## Abstract

The use of conventional antibiotics has substantial clinical efficacy, however these vital antimicrobial agents are becoming less effective due to the dramatic increase in antibiotic-resistant bacteria. Novel approaches to combat bacterial infections are urgently needed and bacteriocins represent a promising alternative. In this study, the activities of the two-peptide bacteriocin PLNC8 αβ were investigated against different *Staphylococcus* spp. The peptide sequences of PLNC8 α and β were modified, either through truncation or replacement of all *L*-amino acids with *D*-amino acids. Both *L*- and *D*-PLNC8 αβ caused rapid disruption of lipid membrane integrity and were effective against both susceptible and antibiotic resistant strains. The *D*-enantiomer was stable against proteolytic degradation by trypsin compared to the *L*-enantiomer. Of the truncated peptides, β1–22, β7–34 and β1–20 retained an inhibitory activity. The peptides diffused rapidly (2 min) through the bacterial cell wall and permeabilized the cell membrane, causing swelling with a disorganized peptidoglycan layer. Interestingly, sub-MIC concentrations of PLNC8 αβ substantially enhanced the effects of different antibiotics in an additive or synergistic manner. This study shows that PLNC8 αβ is active against *Staphylococcus* spp. and may be developed as adjuvant in combination therapy to potentiate the effects of antibiotics and reduce their overall use.

## Introduction

Although antibiotics are the most effective treatment against bacteria of the genus *Staphylococcus* (including the species *S. aureus* and *S. epidermidis*), these opportunistic pathogens are one of the leading causes of severe bacterial infections in humans connected to chronic wounds and medical devices, e.g. catheters and prosthetic implants^1^. These persistent infections are generally difficult to treat, which increases the risk for bacterial dissemination and development of systemic complications^2,3^. Furthermore, considering the gradual increase in antimicrobial resistance, treatment may be even more difficult to achieve as the available options become limited^4^. Consequently, there is an urgent need to find new approaches in human medicine against bacterial infections, and bacteriocins represent a promising avenue that requires more consideration^5,6^.

Bacteriocins are antimicrobial peptides that are produced by most microorganisms that contribute their defence mechanisms. These peptides are divided into class I-V based on their structural characteristics. Class I includes small peptides (<5 kDa) with unusual amino acids, such as lanthionine and β-methyllanthionine that are post-translationally introduced and class II peptides are synthesized in precursor forms and processed (<10 kDa), and includes bacteriocins composed of two peptides (class IIb), such as PLNC8 αβ. Class III bacteriocins are large (>10 kDa) and sensitive to heat, class IV are small (<10 kDa) and circular peptides. Class V are small (<5 kDa), circular or linear peptides that are characterized by containing cross-linkages between cysteine residues and other amino acids, introduced by extensive post-translational modifications^7^. PLNC8 α and β are short peptides, composed of 29 and 34 amino acids, respectively, and show structural stability against heat and pH.

Strains of *Lactobacillus plantarum* are generally recognized as probiotic and are used as dietary supplements, and have been reported to express several bacteriocins that belong to class IIb, including PLNC8 αβ^8^. Since antibiotics are becoming less effective, bacteriocins with antimicrobial activity are attractive candidates in human medicine due to their characteristics of displaying low toxicity towards eukaryotic cells and considered safe and harmless to human, and are active against pathogenic bacteria that have acquired resistance to antibiotics^5,9^. These peptides show structural stability against heat and changes in pH, and bactericidal activity against a wide range of microbes^8,10,11^. Peptides are *in vivo* exposed to various physical, chemical and biological conditions^12^, affecting their activity and bioavailability. We have recently shown that PLNC8 αβ permeabilizes the Gram negative oral pathogen *Porphyromonas gingivalis* and counteracts its cytotoxic and immunomodulatory effects on human cells^13,14^. Bacteriocins may also be used in combination with other antimicrobial agents, e.g. antibiotics, to enhance their effects. Several *Staphylococcus* species have been shown to be synergistically inhibited by a combination of nisin with citric acid^15^ or with traditional antibiotics, including penicillin and chloramphenicol^16^, against several *Staphylococcus* species.

Since infections caused by *Staphylococcus* spp. is one of the most problematic infections in humans, it is important to find alternative treatments consisting of novel antimicrobial compounds. The potency and low toxicity of bacteriocins may potentially reduce the overall use of antibiotics. As a consequence, the development and spreading of antimicrobial resistance may be suppressed by using bacteriocins in combination with low doses of antibiotics. Although bacteriocins retain properties suitable for treatment applications in clinical settings, studies are needed to clarify their mechanism of action and development of resistance^17^. This study aims to investigate the antimicrobial activity of PLNC8 αβ and derivatives of PLNC8 αβ, alone or in conjunction with selected and currently utilized antibiotics, against *Staphylococcus* spp., including human clinical isolates. The ultimate goal is to identify alternative strategies that may be utilized in the future to prevent or treat infections caused by these opportunistic pathogens, and thus reduce the use of traditional antibiotics. We show that bacteriocin PLNC8 αβ has high efficacy against *S. aureus* and *S. epidermidis*, and is able to additively/synergistically reduce the concentrations of conventional antibiotics, demonstrating promising potential for this antimicrobial agent to be further developed for use in clinical settings and health care systems.

## Materials and Methods

### Bacterial strains and culture conditions

The following bacterial strains were used: *S. aureus* CCUG 35601 (MRSA, Culture Collection, University of Gothenburg, resistant against methicillin, gentamicin and tetracycline) and *S. aureus* ATCC 29213 (MSSA, ATCC, Manassas, VA). *S. epidermidis* ATCC 12228 (ATCC, Manassas, VA), RP62A, N15 and 10 clinical isolates of *S. epidermidis* that have previously been characterized^18,19^. Five strains have heterogeneous resistance against the glycopeptide antibiotics vancomycin and teicoplanin. The bacteria were grown on Luria-Bertani (LB) agar plates, supplemented with 6% defibrinated horse blood (Håtunalab AB, National Veterinary Institute, Sweden), and incubated at 37 °C overnight. Single colonies were inoculated into 5 ml of LB broth and the bacteria were allowed to grow overnight at 37 °C on a shaker (300 rpm). Viable count was used to quantify the bacterial concentration, which was adjusted to correspond to 10^9^ CFU/ml.

### Peptide synthesis

**Figure 1:**
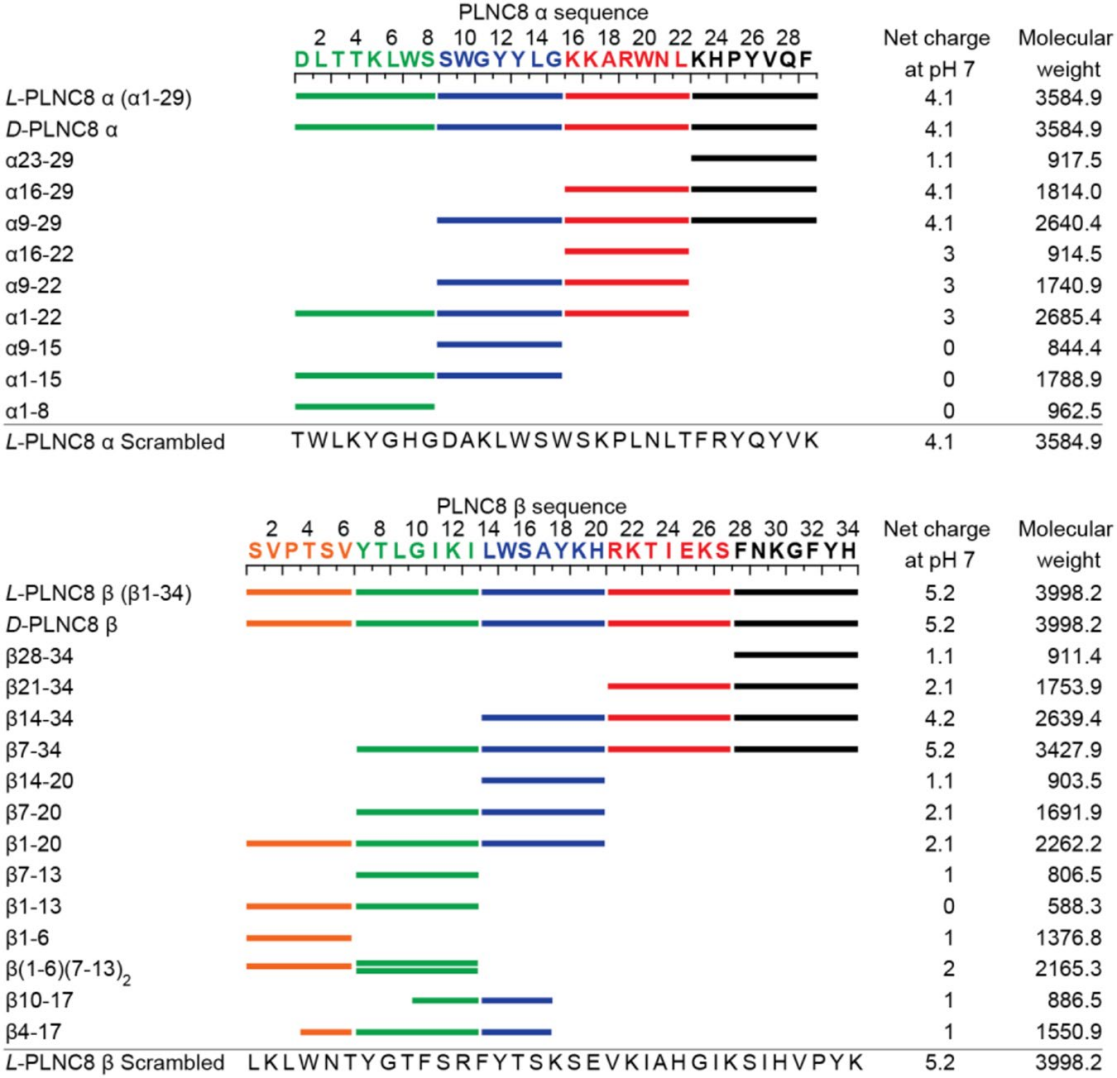
Amino acid sequences, net charge and molecular weight of full-length and truncated forms of PLNC8 αβ peptides.

Peptides were synthesized using an Fmoc approach on a Quartet automated peptide synthesizer (Protein Technologies, Inc). All truncated peptides were synthesized in a 25 µmol scale whereas full length peptides (*L*-, *D*- and scrambled) were synthesized in 100 µmol scale. All peptides were synthesized with free C-terminals (-COOH) and N-terminals (-NH_2_) and the sequences can be found in Fig. 1. A wang resin (Novabiochem, 1.13 mmol/g) was used as solid support for all peptides. Loading of the first Fmoc protected amino acid was accomplished by treating the solid support with a mixture containing Fmoc-amino acid (5 equivalents (eq)), 1-(Mesitylene-2-sulfonyl)-3-nitro-1H-1,2,4-triazole (MSNT, 5 eq) and 1-Methylimidazole (Melm, 3.75 eq) in dry DCM in a N_2_ atmosphere. After 1 hour the resin was filtered off and the loading procedure was repeated. Sequential couplings were performed using 4 eq of amino acid in *L*- or *D*-form and *O*-(Benzotriazol-1-yl)-*N*,*N*,*N*′,*N*′-tetramethyluronium tetrafluoroborate (TBTU) (Iris biothech gmbh) and using 8 eq of diisopropylethylamine as base. Fmoc deprotection of coupled amino acids were performed by exposure to Piperidine (20% in DMF, v/v). Crude peptides were cleaved from their resin and globally deprotected by treatment with a cleavage cocktail containing trifluoroacetic acid (TFA)/water/triisoproylsilane (95:2.5:2.5, v/v/v, 2 hour). The crude peptides were filtered off and concentrated using a flow of N_2_ before being precipitated and washed with cold diethyl ether. Purification of the crude peptides were done on a reversed phase HPLC system (Dionex Ultimate 3000 LC, Thermo Scientific) using a Kromatek HiQ-Sil C18HS column. Final purity was confirmed by analytical HPLC (Supplementary Fig. S1) and MALDI-ToF mass spectrometry (UltraflexXtreme, Bruker Daltonics) (Supplementary Fig. S2).

### Liposome preparation

Liposomes were prepared by thin-film hydration followed by extrusion^20^. The lipids (Merck KGaA, Darmstadt, Germany), 1-palmitoyl-2-oleoyl-sn-glycero-3-phospho-L-serine (POPS), 1-palmitoyl-2-oleoyl-sn-glycero-3-phosphatidylcholine (POPC), 2-Oleoyl-1-palmitoyl-sn-glycero-3-phospho-rac-(1-glycerol) (POPG), and cholesterol, were dissolved in chloroform to a final concentration of 10 mg/ml and mixed to the appropriate ratio. The chloroform was evaporated using a stream of N_2_ and the lipid films were stored in an exicator overnight. The lipid films were hydrated using phosphate buffer (10 mM, pH 7.4) containing a mixture of 90 mM NaCl and 5(6)-carboxyfluorescein (CF, 50 mM) for fluorescence leakage assays and PBS buffer (10 mM PO_4_^3−^, 137 mM NaCl, and 2.7 mM KCl, pH 7.4) for CD measurements. The hydrate lipid films were vortexed for 60 sec and the incubated for 1 hour on an orbital shaker. The liposomes were extruded 21 times through a polycarbonate membrane with 100 nm pores and purified using a PD minitrap G-25 gel column (GE Healthcare, Uppsala, Sweden) running with PBS as eluent.

### Carboxyfluorescein (CF) release assay

Disturbing membrane integrity of liposomes containing self-quenching concentrations of CF results in an increased fluorescence signal due to CF leakage. CF-loaded liposomes (total lipid concentration, 25 µM in PBS) were subjected to antimicrobial peptides (0.005–100 µM). The peptides were added alone or in combinations and the samples were incubated for 30 min before commencing measurements. The CF-release was measured using an extinction wavelength of 492 nm and an emission wavelength of 517 nm using a fluorescence plate reader (Safire 2, Tecan, Austria). All samples were background subtracted using the fluorescence signal prior to peptide addition. The maximum release (100% release) was estimated after peptide mediated CF-release measurements by adding 1% Triton X-100 and incubating for at least 10 min to establish the maximum intensity of the fluoresce signal. CF-release data were fitted to a sigmoidal monophasic (Hill 1) equation and the concentration of peptides needed to produce a 50% CF-release were extracted.

### Circular dichroism (CD) spectroscopy

Antimicrobial peptides typical resides as a random coil in solution but undergo a structural reorganization when interacting with a lipid membrane^21^. Circular dichroism spectroscopy measurements were performed on a Chirascan (Applied Photophysics, United Kingdom) using a 1 mm cuvette at room temperature. A wavelength scan of 195–280 nm was recorded 3 times for each sample, averaged and baseline corrected using PB buffer (pH 7.4, 10 mM). In all samples, the concentration of each peptide was 30 μM, prepared in PB buffer. In experiments with liposomes the final lipid concentration was 660 μM (0.5 mg/ml). To compensate for the different total peptide concentrations used, the averaged data were converted to mean residue ellipticity (MRE).

### Proteolytic degradation

Full length PLNC8 αβ (100 μM) in both *L*-and *D-*form was subjected to Trypsin (0.125 mg/ml, ~5 μM) in ammonium bicarbonate buffer (50 mM, pH 8.5) for 16 hours at 37 °C. Sample solutions were acidified by adding 2.5% TFA and dried in an exicator at room temperature. Samples were resuspended in MQ-water containing 0.1% TFA, desalted using ZipTip-C18 columns (Millipore) and analyzed using MALDI-ToF with α-cyano-4-hydroxycinnamic acid as matrix.

### Haemolysis and cytotoxicity

The haemolytic activity of the peptides was investigated by collecting blood from healthy volunteers in heparinized vacutainers. Briefly, the blood was centrifuged at 600 × g for 5 min and the erythrocyte pellet was washed three times in PBS. The cells were then suspended in PBS and added to 96-well plates (15% erythrocyte suspension/well), containing the peptides with two-fold serial dilution. The plates were incubated for 1 h at 37 °C followed by centrifugation for 5 min at 900 × g and measurement of the supernatants at 540 nm. Haemolytic activity (%) was calculated by subtracting the negative control from all values and normalization against the positive control (0.5% Triton X-100), that was set to 100%. All experiments, each in duplicate, were repeated three times.

Human keratinocytes (HaCaT, CLS Cell Lines Services, 300493) were grown in Dulbecco´s Modified Eagle Medium (DMEM) with 10% fetal bovine serum (FBS, Invitrogen Ltd, Paisley, UK). The cells were maintained at 95% air, 5% CO_2_ and 37 °C and then seeded in 24-well plates at a density of 5 × 10^4^ cells/well and incubated overnight. The medium was replaced with fresh, pre-warmed medium before the cells were exposed to different concentration of PLNC8 αβ for 24 h. The supernatants were collected and Lactate dehydrogenase (LDH) activity (Life Technologies, Stockholm), was measured according to the manufacturers’ protocol.

### Aggregation and ATP release

Aggregation and extracellular release of ATP were used to study the effects of PLNC8 αβ on the bacteria. ATP was registered using a luciferin/luciferase bioluminescence assay (Sigma, St. Louis, Mo, USA) in bacterial suspensions (2.5 × 10^8^ CFU/ml). Bacterial suspensions were exposed to various concentrations of PLNC8 αβ, and real-time changes in light transmission and bioluminescence was registered in a Chronolog lumi-aggregometer (Chrono-Log, Haverton, PA, USA) for 30 min. Secreted ATP levels were analysed by comparing the bioluminescence response with the signals obtained with known concentrations of ATP. Three independent experiments were performed.

### Microscopy

The fluorescent dye Sytox® Green was used to investigate membrane permeabilization caused by PLNC8 αβ. The advantage of this fluorophore is that it can only cross damaged membranes and fluoresce upon binding to nucleic acids. *S. epidermidis* was washed and resuspended in Krebs-Ringer buffer (KRG) (120 mM NaCl, 4.9 mM KCl, 1.2 mM MgSO_4_, 1.7 mM KH_2_PO_4_ and 8.3 mM Na_2_HPO_4_, pH 7.3) incubated for 2 min with or without peptides in 96-well microtiter plates. Images were captured with Olympus BX41 and the fluorescence intensity was analysed and quantified using the software ImageJ. Electron microscopy was used to visualize the damage of *S. epidermidis* that is caused by full-length and truncated peptides of PLNC8 αβ. Briefly, the bacteria were pelleted and washed with KRG followed by exposure to different peptide combinations at a final concertation of 25 µM for 5 min, followed by fixation in 2.5% glutaraldehyde in 0.1M phosphate buffer, pH 7.3. For transmission electron microscopy (TEM), samples were washed in 0.1M phosphate buffer and postfixed in 2% osmium tetroxide in 0.1M phosphate buffer for 2 h and embedded into LX-112 (Ladd, Burlington, Vermont, USA). Ultrathin sections (approximately 50–60 nm) were cut by a Leica ultracut UCT/Leica EM UC 6 (Leica, Wien, Austria). Sections were then contrasted with uranyl acetate followed by lead citrate and examined in a Hitachi HT 7700 (Tokyo, Japan). Digital images were taken by using a Veleta camera (Olympus Soft Imaging Solutions, GmbH, Münster, Germany). For scanning electron microscopy, specimens were fixed as described above and briefly rinsed in distilled water. The specimens were dehydrated in 70%, 95% and absolute ethanol for 10 min each and finally put into acetone for 10 min, and then dried in a critical point dryer (Balzer, CPD 010, Lichtenstein) with carbon dioxide. After drying, specimens were mounted on an aluminum stub and coated with Carbon (Bal-Tec Med 010, Lichtenstein), and analyzed in an Ultra 55 field emission scanning electron microscope (Zeiss, Oberkochen, Germany) at 3 kV.

### Antimicrobial activity

The broth microdilution method was used to determine minimal inhibitory concentration (MIC) and minimal bactericidal concentration (MBC). Briefly, two-fold serial dilutions of the peptides were used and the final concentrations ranged between 0.097–50 µM. The final concentrations of the antibiotics vancomycin and teicoplanin ranged between 0.097–50 µg/ml, while rifampicin ranged between 0.0019–1 µg/ml and gentamicin 0.0097–5 µg/ml. The inhibitory and bactericidal effects of peptides together with antibiotics were studied by using the same concentration series of antibiotics with constant, sub-MIC concentrations, of the peptides in all the wells (see figure legends). The plates were then incubated at 37 °C for 20 h. Visual inspection and spectroscopical quantification (620 nm) were used to determine the MIC as the lowest concentration that completely inhibited bacterial growth. MBC was determined as the lowest concentration where no growth of bacterial colonies (10 µl) was observed on blood-agar plates. All experiments were repeated at least three times. The combined effect of teicoplanin and PLNC8 αβ against *S. epidermidis* ATCC 12228 was evaluated by the microdilution checkerboard method. Briefly, two-fold serial dilutions of the antibiotics and PLNC8 αβ were prepared together in microtiter plates, either alone or in combination. The MIC values were determined visually and spectroscopically, and the fractional inhibitory concentration (ΣFIC) was calculated by the equation (MIC of PLNC8 αβ in combination/MIC of PLNC8 αβ alone) + (MIC of antibiotic in combination/MIC of antibiotic alone). Synergy was defined as ΣFIC ≤ 0.5, additive when 0.5 <ΣFIC ≤1, indifferent when 1 <ΣFIC <2 and antagonistic when ΣFIC ≥ 2.

Furthermore, a gel with a final concentration of 100 µM *L*-PLNC8 αβ was prepared. The gel contained 10% glycerol (Sigma Aldrich, ≥99.5%) and 10% gelatine (Sigma Aldrich, from cold water fish skin, 45%). The antimicrobial activity of the peptides in this formula was investigated in a fluorescence microscope with Sytox Green and on agar plates. Briefly, *S. epidermidis* ATCC 12228 was spread on agar plates and allowed to dry, followed by addition of a 5 µl drop of control gel or gel containing the peptides. A plastic loop was used to spread the drop to obtain a gradient. The plates were incubated at 37 °C for 20 h.

### *In vitro* resistance study

*S. aureus* were cultured in LB broth at 37 °C with constant shaking at 200 rpm for 20 h, and sub-cultured daily by inoculating 20 µl bacterial suspension into 2 ml LB broth. After 5 transfers, 20 µl of bacterial suspension was inoculated into 2 ml LB broth without (control) or with *L*-PLNC8 αβ at a final concentration of 1.5 µM, for 10 transfers. The treated bacteria were then transferred to broth containing *L*-PLNC8 αβ at a final concentration of 6.25 µM for 10 passages. Susceptibility testing was performed for all passages, using the broth microdilution method as described above.

### Surface-associated bacteria

*S. epidermidis* RP62A was inoculated into 5 ml of LB broth and incubated on a shaker at 37 °C overnight. The bacterial culture was diluted 1:100 into fresh media and 100 µl of bacterial suspension per well was added in a 96-well microtiter plate and incubated statically at 37 °C for 20 h. The wells were washed three times by submerging the plate into a container with distilled water to remove unattached cells. Fresh LB media was added to each well (100 µl) followed by addition of the peptides in different concentrations. The plate was incubated statically for 1 h. Detached material in the wells were transferred to a new microtiter plate for absorbance measurements at 620 nm. The remaining attached bacteria were stained with 0.1% crystal violet for 15 min before the plate was washed four times in distilled water as mentioned above and allowed to dry at room temperature for 2 h. The crystal violet was solubilized in 30% acetic acid for 15 min and the absorbance quantified at 540 nm. Each experiment, with three replicates, was repeated three times.

### Ethics statement

This work deals with clinical bacterial isolates from human infections. No tissue material or other biological material was stored from the patients, only sub-cultured bacterial isolates. Swedish law does not require ethical approval for work with bacterial isolates from humans. All information regarding these isolates was anonymized. Ethical permission for collecting heparinized blood from healthy volunteers was approved by the regional ethical board at Örebro-Uppsala County (Dnr 2015/543). Informed consent was obtained from all volunteers. Collection of blood and associated methods were carried out in accordance with relevant guidelines and regulations.

## Results

### Antimicrobial activity of native *L*- and *D*-PLNC8 αβ

We have recently shown that PLNC8 αβ is a potent antimicrobial agent against the Gram-negative bacteria *P. gingivalis*^13,14^. The purpose of this study was to determine the antimicrobial activity of PLNC8 αβ against susceptible and antibiotic-resistant strains of staphylococci.

Peptides are constantly subjected to proteolytic degradation in biological systems. In order to investigate the stability of PLNC8 αβ enantiomers against proteolytic degradation, the peptides were treated with trypsin for 16 h. The molecular weight of the peptides, before and after trypsin treatment, was determined using matrix assisted laser dissociation and ionization time of flight mass spectrometry (MALDI-ToF MS). Both PLNC8 α and β of the *L*-form, but not the *D*-form, were efficiently degraded by trypsin (Supplementary Fig. S3). Among the degradation products of *L*-PLNC8 β, the amino acid sequences of 1–21, 1–22 and 1–26 were found to be accumulated. Interestingly, these fragments of PLNC8 β were also shown to exhibit inhibitory effects on their own (see below).

**Figure 2:**
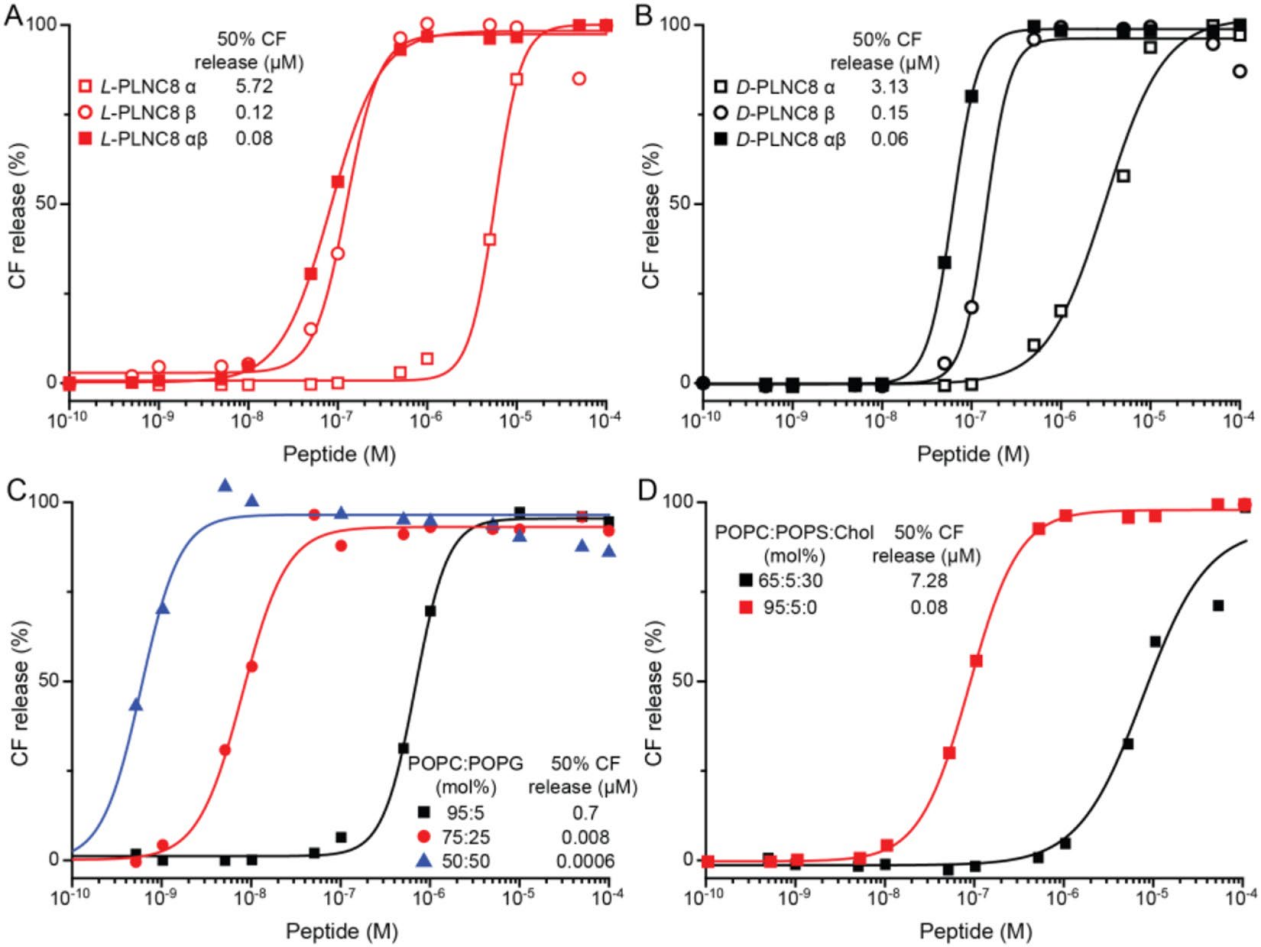
Permeabilization of model lipid membranes by PLNC8 α and β. Release of (6)-carboxyfluorescein (CF) was recorded after exposure of liposomes composed of POPC:POPS (95:5) with (**A**) *L*-PLNC8 α, β and αβ or (**B**) *D*-PLNC8 α, β and αβ. CF-release from model systems containing 5, 25 or 50% POPG (**C**) or containing cholesterol (**D**) due to exposure to *L*-PLNC8 αβ.

The membrane activity of PLNC8 α and β, alone and when combined, was evaluated using liposomal model systems with different ratios of the zwitterionic lipid POPC and the negatively charged lipids POPS and POPG, with and without cholesterol. In liposomes comprised of POPC and POPS (95:5), 0.12 μM PLNC8 β triggered 50% release of encapsulated carboxyfluorescein (CF) after 30 min whereas only limited release was seen in the presence of PLNC8 α (Fig. 2A,B). The combination of PLNC8 α and β in a molar ratio of 1:1 showed efficient permeabilization of the liposomes and both the *L*- and the *D*-enantiomers of the peptides exerted similar activity, resulting in 50% release of CF at 0.08 and 0.06 µM for *L*- and *D*-PLNC8 αβ, respectively. To investigate the effect of the lipid composition, various ratios of POPC and POPG were evaluated. Whereas POPS can be used to mimic the negative net charge of bacterial membranes, POPG is a more common lipid in bacterial membranes^22^. At a ratio of POPC:POPG (95:5) the lipid membrane was slightly more resilient to perturbation by *L*-PLNC8 αβ compared to POPS containing lipid membranes, requiring a peptide concentration of 0.7 µM to reach 50% CF-release (Fig. 2C). This is likely a result of the slightly higher negative net charge of POPS. Exposing the liposomes to the individual peptides resulted in a similar trend. Peptide concentrations of 0.12 and 5.7 µM for PLNC8 β and PLNC8 α, respectively, were required in order to produce a 50% CF-release (Fig. S4A,B). Increasing the ratio of POPG further to 25–50% made the liposomes even more susceptible to the peptides, corresponding to a 100–1000 fold increase in efficiency (Fig. 2C). The individual peptides displayed a similar trend (Fig. S4A,B), although the effect on lipid composition was not as pronounced for PLNC8 α as for PLNC8 β. Liposome net charge is consequently a central aspect in regulating the membrane activity of the peptides. To investigate the potential influence of cholesterol on the permeabilizing effect of PLNC8, a model system comprising POPC:POPS:Cholesterol (65:5:30) was used. In the presence of cholesterol, a 100-fold higher concentration of PLNC8 αβ was required to trigger a similar CF release as in the absence of cholesterol (Fig. 2D). The same trend was observed for the individual peptides were the membrane activity of PLNC8 α was almost abolished in the presence of cholesterol (Fig. S4A,B). Cholesterol can thus protect eukaryotic cells from the lipid membrane perturbating effects of PLNC8.

**Figure 3:**
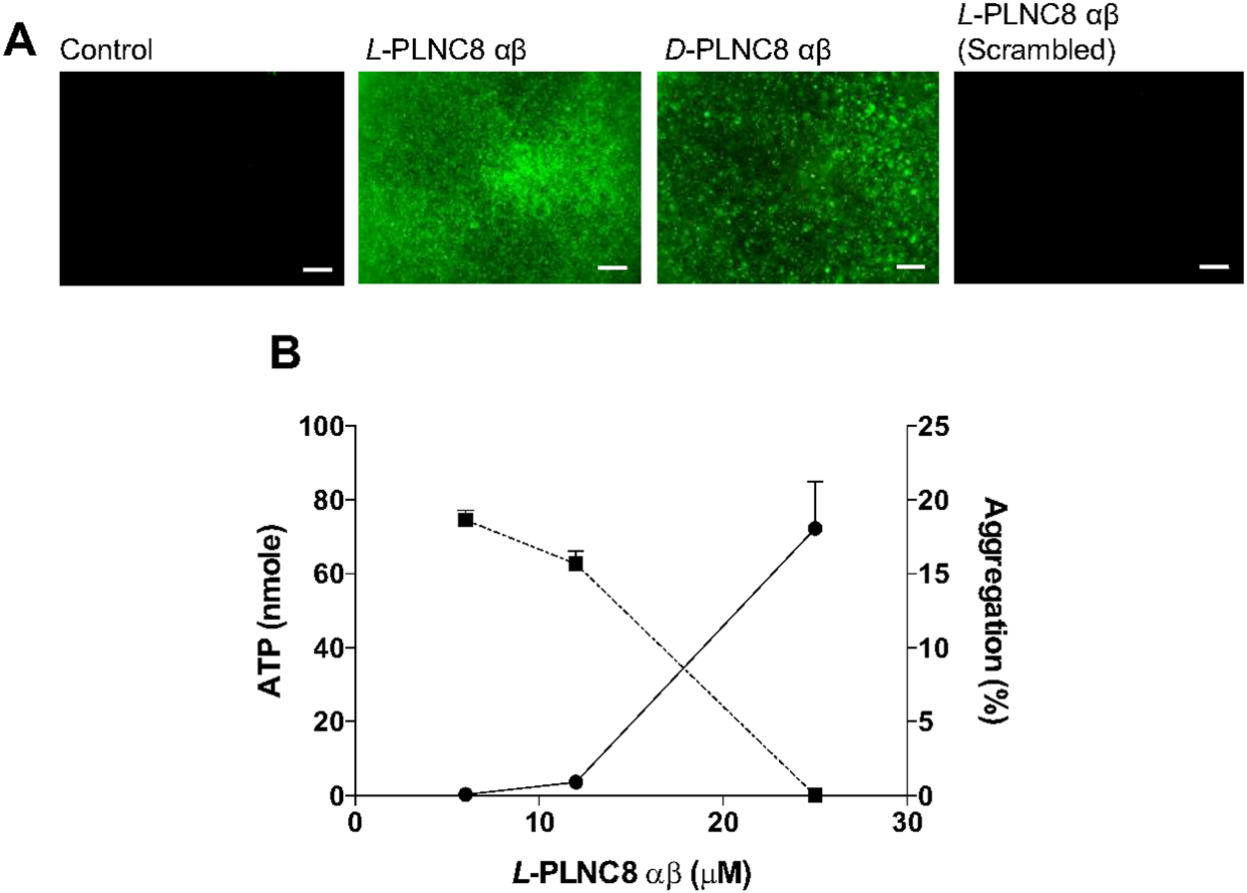
Rapid bacterial permeabilization by PLNC8 αβ. (**A**) Uptake of Sytox Green by *S. epidermidis* ATCC 12228 after exposure to 5 µM of *L*-PLNC8 αβ, *D*-PLNC8 αβ or scrambled-PLNC8 αβ for 2 min, scale bar = 300 µm. (**B**) Aggregation (dotted line) and ATP release (solid line) were recorded to determine bacterial permeabilization by PLNC8 αβ. Low PLNC8 αβ concentration caused bacterial aggregation with minor lysis, while higher PLNC8 αβ concentration caused rapid and efficient permeabilization with no aggregation. Data from three independent experiments are presented as mean with standard error of the mean (SEM).

The antimicrobial effects of both PLNC8 αβ enantiomers were rapid as indicated by the enhanced uptake of Sytox Green already after 2 min of treatment (Fig. 3A). When scrambling the peptide sequence, no bacterial membrane permeabilization was obtained, which indicate that the membrane interactions are likely folding dependent. The rapid lytic effects of PLNC8 αβ was further demonstrated on liposomes (POPC:POPS (95:5)), where PLNC8 β and PLNC8 αβ (1:1), but not PLNC8 α, caused complete lysis after 2 min (Supplementary Fig. S4). The same concentration dependent rapid permeabilization was observed for all liposomal model systems investigated (data not shown). Bacterial permeabilization by PLNC8 αβ was further verified by measuring aggregation and ATP release (Fig. 3B). At low concentrations (≤6.25 µM), PLNC8 αβ caused bacterial aggregation with minor ATP release. Increasing concentrations of PLNC8 αβ resulted in less bacterial aggregation while permeabilization was considerably enhanced, as revealed by a rapid (seconds) release of extracellular ATP.

Antimicrobial peptides tend to adopt a more defined secondary structure when interacting with lipid membranes. The structural changes of PLNC8 αβ when interacting with a lipid model system composed of POPC:POPS (95:5) was determined using circular dichroism (CD) spectroscopy. CD spectra of both enantiomers of PLNC8 α and β, separately and when combined, showed induced structural changes when combined with the liposome model system (Supplementary Fig. S6). The enantiomers did, as expected, show mirrored spectra because of the opposite chirality of the peptides. Enantiomers of PLNC8 α only displayed minor structural changes while PLNC8 β showed a more pronounced change in structure, from random coil to a helical structure, when interacting with the liposomes. Interestingly, previous studies have indicated that PLNC8 β can adopt a β-sheet structure when interacting with liposomes with the same lipid composition but with both constituents at higher concentrations^14^, illustrating a structurally highly dynamic system. When combining both peptides (PLNC8 αβ), a large structural rearrangement was seen, which due to the spectral contributions from both peptides, is difficult to qualitatively and quantitatively define. In addition, PLNC8 αβ also showed tendencies to cause aggregation of the liposomal model system seen as a slight decrease in the CD intensity.

Both *L*- and *D*-enantiomers of PLNC8 αβ were tested for their antimicrobial activity on *S. epidermidis*. The individual peptides showed generally poor activity on *S. epidermidis*, while the combination of α and β resulted in a pronounced antimicrobial effect (data not shown). The inhibitory (MIC) and bactericidal (MBC) concentrations of the peptides on *S. epidermidis* were 6.25 and 12.5 µM, respectively, for the *L*-enantiomer, and 12.5 µM for the *D*-enantiomer.

PLNC8 αβ belongs to class IIb bacteriocins that consist of two separate peptides. Optimal antimicrobial activity is dependent on the complementary action of the two peptides. It is therefore necessary to determine the optimal molar ratio between PLNC8 α and β against *Staphylococcus*. While the total concentration of both peptides was kept constant, the concentrations of *L*-PLNC8 α and *L*-PLNC8 β, respectively, were varied to obtain different molar ratios. Furthermore, since *L*-PLNC8 β alone was shown to permeabilize both liposomes and bacteria, the concentration of *L*-PLNC8 α was decreased from 50 to 1.5 µM whereas *L*-PLNC8 β was increased from 50 to 98 µM while keeping the total concentration of peptides at 50 µM, followed by two-fold serial dilutions. Optimal inhibitory and bactericidal activity of *L*-PLNC8 αβ against *S. epidermidis* ATCC was achieved at a molar ratio of 1:1. The effects decreased when the ratio was altered towards higher concentration of β (Table 1). Subsequent experiments in the study were thus performed with a molar ratio of 1:1 (α:β).

**Table 1:** The molar ratio of PLNC8 α and β is critical for optimal antimicrobial activity.

Ratio *L*-PLNC8 (α:β)*	MIC (µM)	MBC (µM)
1:1	6.25	12.5
1:3	6.25	25
1:7	12.5	50
1:15	50	>50
1:31	50	>50
1:64	50	>50

*S. epidermidis* ATCC 12228 was exposed to different molar ratios of *L*-PLNC8 α and β for 20 h. A molar ratio of 1:1 between *L*-PLNC8 α:β is most efficient at inhibiting and killing *S. epidermidis*. MIC and MBC of different molar ratios of *L*-PLNC8 α and β. *The highest total concentration of the peptides was kept constant at 50 µM, while the concentrations of *L*-PLNC8 α and β was individually altered to obtain different molar ratios. All experiments were performed three times.

In order to further verify the antimicrobial activity of *L*-PLNC8 αβ, the effects in a number of different *Staphylococcus* strains were examined. *L*-PLNC8 αβ was found to target the bacteria with similar effects, irrespective of the characteristics of the bacteria, including antibiotic resistance (MRSA vs MSSA) and ability to form biofilms (Table 2A). Although *S. epidermidis* was more susceptible than *S. aureus*, the recorded MIC and MBC concentrations were generally comparable between the different strains.

**Table 2:** Susceptibility of *Staphylococcus* to PLNC8 αβ.

A	Description	*L*-PLNC8 αβ (µM)			
Bacteria		MIC		MBC	
*S. aureus* ATCC 29213 (MSSA)	Methicillin sensitive	12.5		25	
*S. aureus* CCUG 35601 (MRSA)	Methicillin resistant	12.5		25	
*S. epidermidis* ATCC 12228	Biofilm negative	6.25		12.5	
*S. epidermidis* RP62A	Biofilm positive	6.25		6.25	
*S. epidermidis* N15	Nose of a healthy individual	6.25		6.25	
*S. epidermidis* 117	Infected hip joint prosthesis	6.25		12.5	
**B**	**Passages**	***L*-PLNC8 αβ (µM)**		***D*-PLNC8 αβ (µM)**	
**Bacteria**		**MIC**	**MBC**	**MIC**	**MBC**
*S. aureus* ATCC (Control)	20	12.5	25	12.5	25
*S. aureus* ATCC (Treated)	20	12.5	25	12.5	25

(**A**) Different *Staphylococcus* species were cultures for 20 h in the presence of increasing concentrations of *L*-PLNC8 αβ (1:1). *S. epidermidis* was more susceptible to *L*-PLNC8 αβ than *S. aureus*. MIC and MBC of different *Staphylococcus* species in response to *L*-PLNC8 αβ. All experiments were performed three times. (**B**) *S. aureus* were grown in suspension in the presence or absence of sub-MIC concentrations of *L*-PLNC8 αβ (1.5 µM for 10 passages and 6.25 µM for 10 passages). Susceptibility of *S. aureus* was determined for all passages. The table shows MIC and MBC values from the last passage.

Development of bacterial resistance against *L*-PLNC8 αβ was investigated after exposure of *S. aureus* to sub-MIC concentrations of the peptides for 20 passages. The susceptibility of *S. aureus* towards *L*- and *D*-PLNC8 αβ was not altered as the inhibitory and bactericidal concentrations remained unchanged at 12.5 µM and 25 µM, respectively (Table 2B).

### Effect of PLNC8 αβ on attached bacteria

**Figure 4:**
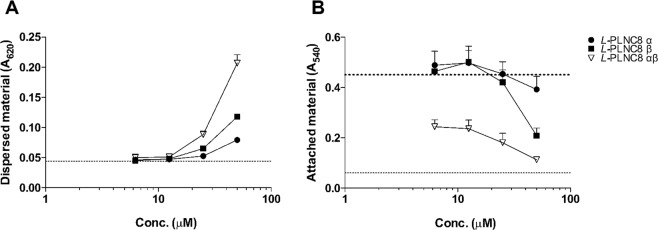
PLNC8 αβ is effective against surface-associated *S. epidermidis*. *S. epidermidis* RP62A were allowed to adhere followed by removal of suspended bacteria and addition of the peptides for 1 h. (**A**) Absorbance measurement of detached material. The dotted line indicates the baseline (LB broth). (**B**) Crystal violet (CV) staining of the remaining attached bacterial material. The lower dotted line is the negative control (LB broth) and the upper dotted line is the positive control (untreated bacterial biofilm). Results from three independent experiments are presented as mean with SEM.

Several species of the genus *Staphylococcus* are common pathogens in nosocomial infections associated with their ability to form biofilms and persist on medical devices, such as catheters and medical implants. The concentrations of traditional antibiotics required to treat bacterial biofilms are in the range of 100–1000-fold higher compared to bacteria in suspension, which may cause severe complications during treatment^23^. We show rapid disruption of attached bacterial cells by *L*-PLNC8 αβ (Fig. 4). *L*-PLNC8 α alone caused minor effects, while *L*-PLNC8 β caused substantial disruption at the highest concentration. *L*-PLNC8 α and β together were most effective and caused rapid and dose-dependent disruption of surface-associated *S. epidermidis*.

### Sequence and length optimization of PLNC8 αβ

**Figure 5:**
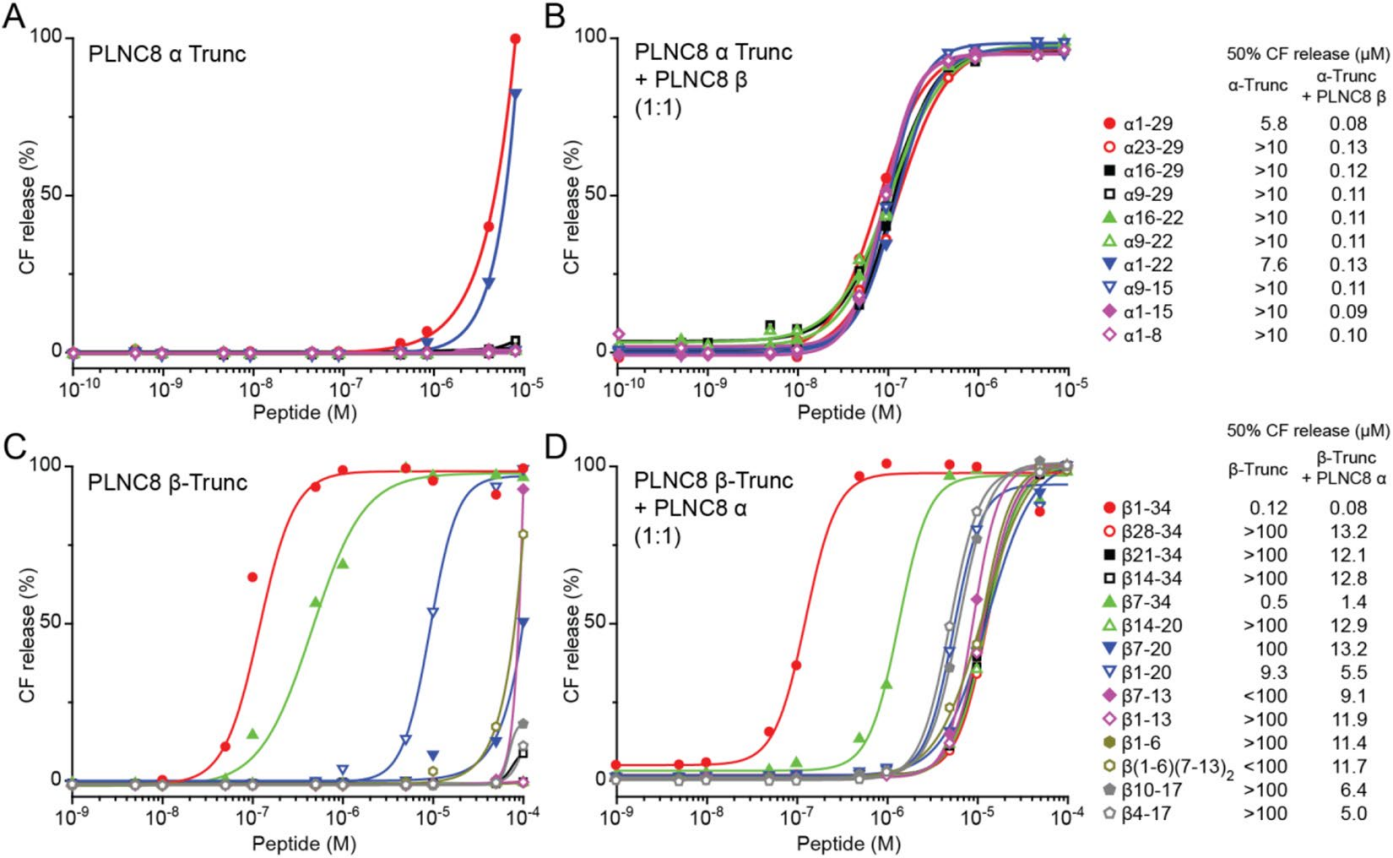
Permeabilizing activity of truncated forms of *L*-PLNC8 α and β. (**A**) CF release from liposomes was obtained with α1–29 (full length) and α1–22. (**B**) Addition of full length *L*-PLNC8 β potentiated the effects of different truncated *L*-PLNC8 α peptides. (**C**) CF release from liposomes was obtained with β1–34 (full length), β7–34, β1–20 and β7–20. (**D**) Addition of full length *L*-PLNC8 α potentiated the effects of different truncated *L*-PLNC8 β peptides. Quantification of 50% CF release by truncated *L*-PLNC8 α or β peptides, with and without *L*-PLNC8 α or β are indicated, n = 3.

Truncated versions of *L*-PLNC8 α and β were investigated with respect to membrane activity and antimicrobial activity using both liposomes and *S. epidermidis*. Among the truncated forms of *L*-PLNC8 α, the amino acid sequence of 1–22 was found to retain a lytic activity in the liposome model systems that was comparable to the full length α-sequence (Fig. 5A). Addition of full-length *L*-PLNC8 β together with the different truncated α-peptides showed only minor differences compared to the effects of full-length *L*-PLNC8 α and β (Fig. 5B).

In addition to full length *L*-PLNC8 β, several of the truncated β-peptides were membrane active and considerably enhanced release of liposome encapsulated CF, including truncated-β7-34, β7-20 and β1-20 (Fig. 5C). Combination of the different truncated *L*-PLNC8 β peptides with full length *L*-PLNC8 α enhanced their activity in the liposome model, with truncated-β7-34 and β7-20 showing the most pronounced effects (Fig. 5D). The amino acid sequences of full length and truncated forms of *L*-PLNC8 α and *L*-PLNC8 β are illustrated in Fig. 1, and the required concentrations for 50% CF release from liposomes are indicated (Fig. 5).

The antimicrobial effect of full length and truncated-α1–22 against *S. epidermidis* could only be observed in the presence of full length *L*-PLNC8 β (Table 3A). However, truncated β-peptides displayed inhibitory activity against *S. epidermidis*, which was similar to the results obtained with the liposome model, i.e. truncated-β7–34, β7–20 and β1–20 inhibited bacterial growth (Table 3B). Furthermore, combination of truncated β-peptides with full length *L*-PLNC8 α enhanced the inhibitory activity of truncated-β7–34. Our results prompted us to combine the active sequences of both truncated *L*-PLNC8 α and truncated PLNC8 β to determine their antimicrobial effects on *S. epidermidis*. Although the peptides were able to efficiently inhibit bacterial growth, especially the combination α1–22/β1–20, they did not show any bactericidal activity (Table 3C).

**Table 3:** Antimicrobial activity of truncated forms of PLNC8 αβ.

Peptide	MIC	MBC	MIC+β	MBC+β	Peptide	MIC	MBC	MIC+α	MBC+α
**A**					**B**				
α23–29	>50	>50	>50	>50	β28–34	>50	>50	>50	>50
α16–29	>50	>50	>50	>50	β 21–34	>50	>50	>50	>50
α9–29	>50	>50	50	>50	β14–34	>50	>50	>50	>50
α1–29	>50	>50	6.25	12.5	β7–34	50	>50	12.5	25
α16–22	>50	>50	>50	>50	β14–20	>50	>50	>50	>50
α9–22	>50	>50	50	>50	β7–20	50	>50	25	>50
α1–22	>50	>50	25	50	β1–20	50	>50	12.5	>50
α9–15	>50	>50	50	>50	β1–34	50	>50	6.25	12.5
α1–15	>50	>50	>50	>50	β7–13	>50	>50	>50	>50
α1–8	>50	>50	>50	>50	β1–6	>50	>50	>50	>50
					β1–13	>50	>50	>50	>50
					β(1–6)(7–13)_2_	>50	>50	>50	>50
					β10–17	>50	>50	>50	>50
					β4–17	>50	>50	>50	>50
**Peptide (1:1)**				**MIC (µM)**			**MBC (µM)**		
**C**									
α1–22/β1–20				12.5			>50		
α1–22/β7–20				25			>50		
α1–15/β1–20				25			>50		
α1–15/β7–20				25			>50		

MIC and MBC values of truncated *L*-PLNC8 α and β against *S. epidermidis* ATCC 12228. (**A**) α1–29 (full-length) and truncated α1–22 showed both inhibitory and bactericidal activity when combined with full-length PLNC8 β. (**B**) β1–34 (full length) β7–34, β1–20 and β7–20 inhibited bacterial growth, while bactericidal effect was obtained when combining β1–34 (full length) and β7–34 with PLNC8 α. (**C**) The combination α1–22/β1–20 was most efficient at inhibiting bacterial growth, however all the combinations lacked bactericidal activity.

Neither the full-length peptides of both *L*- and *D*-enantiomers, nor the truncated α1–22, α1–15, β7–34, β7–20 and β1–20 were cytotoxic at relevant concentrations (<50 µM), as determined by haemolytic activity on isolated human erythrocytes (Supplementary Fig. S7A–D). A concentration of 200 µM of *L*-PLNC8 αβ caused 9.8% erythrocyte cell lysis after 1 hour of incubation.

Cytotoxicity of full length *L*-PLNC8 αβ was evaluated on human keratinocytes and quantified by measuring LDH activity after stimulation with the peptides for 24 h. The peptides showed no cytotoxic effects and the cells exhibited normal morphology compared to the untreated control (Supplementary Fig. S7E).

### PLNC8 αβ permeabilized bacteria and caused morphological changes

**Figure 6:**
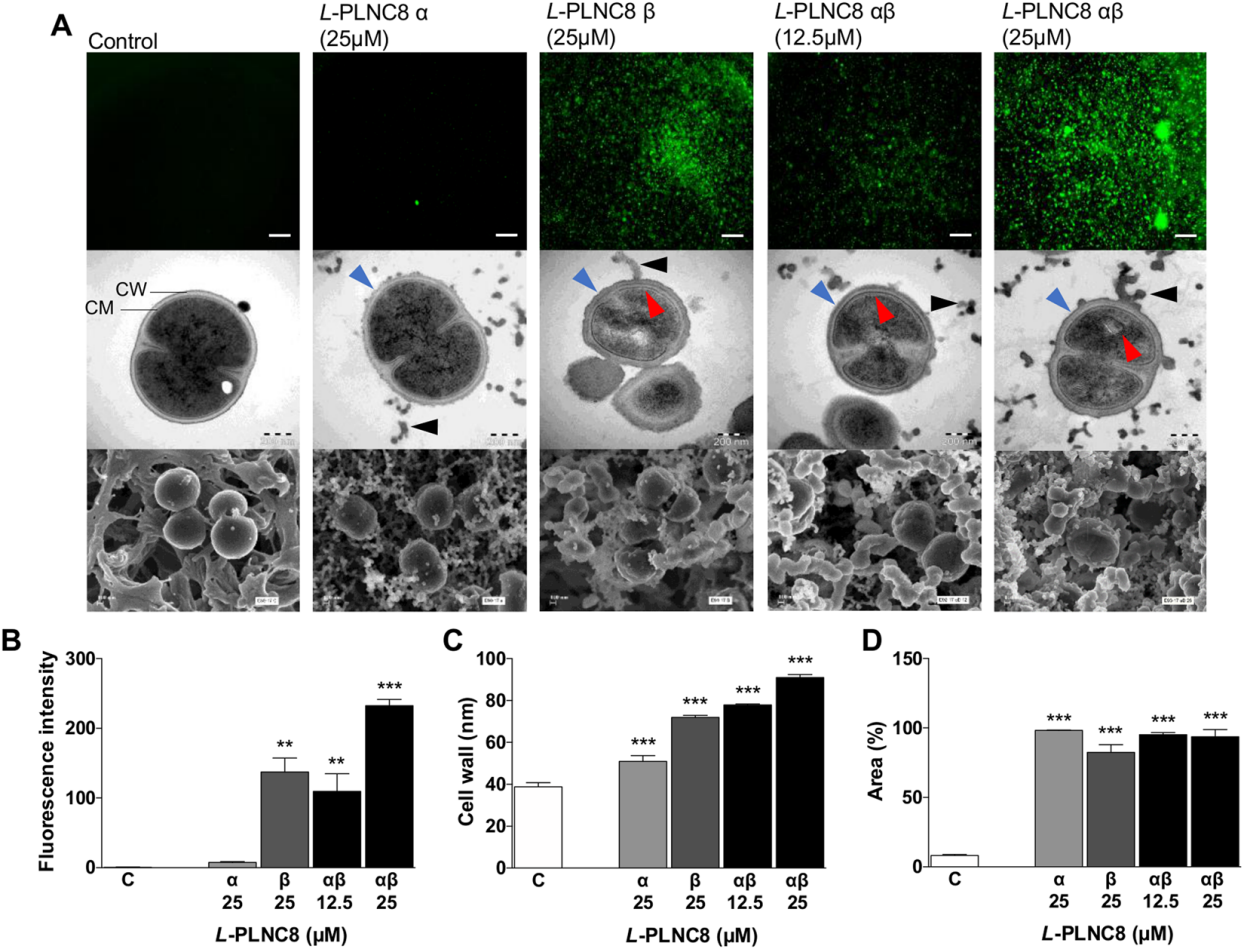
PLNC8 αβ damages the integrity of bacterial cell wall and cell membrane. (**A**) The uptake of Sytox Green by *S. epidermidis* ATCC 12228 after exposure for 5 min to different peptide combinations (25 µM, except for PLNC8 αβ where two concentrations were used, 12.5 µM and 25 µM), indicates damaged cell membrane, scale bar is 300 µm. Visualization of bacterial damage with transmission electron microscopy. The cell membrane (CM) was completely detached (red arrow heads) and leakage of intracellular content is observed (black arrow heads). The thickness of the cell wall (CW) was increased (blue arrow heads). Scale bar is 200 nm. Scanning electron microscopy demonstrates further the substantial amounts of leaked material from the bacteria, particularly by PLNC8 α, while PLNC8 β and the combination of both peptides results in bacterial lysis. Scale bar is 100 nm. (**B**) Quantification of the fluorescence intensity of Sytox Green. (**C**) Quantification of the thickness of the cell wall. (**D**) Quantification of the area covered by bacteria and leaked material.

The cause of the remarkably rapid antimicrobial effect of the two-peptide bacteriocin PLNC8 αβ on *S. epidermidis* shown in the Sytox Green experiments (Fig. 3A) was further investigated using both transmission and scanning electron microscopy. Planktonic bacteria were exposed to the peptides for 5 min followed by fixation and analysis. The bacteria in the untreated sample were intact as indicated by the absence of Sytox Green fluorescence (Fig. 6A). The corresponding electron micrographs also showed normal bacterial cell morphology, where the cell wall and cell membrane could clearly be distinguished in the TEM image. Although *L*-PLNC8 α did not appear to affect cell membrane integrity, as no Sytox Green fluorescence was detected, the electron micrographs revealed a substantial amount of bleb-formation and secretion of micro vesicles that formed complex networks and bacterial aggregation. The effects of *L*-PLNC8 β were, however, distinct and caused rapid and severe morphological changes. The cell wall appeared swollen and the cell membrane was irregular, and consequently, large quantities of intracellular material leaked out and the bacterial size was considerably reduced. Exposure of *S. epidermidis* to *L*-PLNC8 αβ at a 1:1 molar ratio caused severe damage. Formation of large aggregates of collapsed bacteria was observed, which was detected by the fluorescent stain Sytox Green, and the effect of *L*-PLNC8 αβ was concentration-dependent. Besides the apparent effect of a swollen cell wall, the inner cell membrane was completely detached and no longer associated with the inner cell wall zone. Overview images of electron microscopy show the large amount of PLNC8 αβ-induced secretion of bacterial material and formation of large aggregates (Supplementary Fig. S8).

Furthermore, the effects of the truncated peptides β7–20 and β1–20, alone or in combination with full length *L*-PLNC8 α, were visualized by fluorescence microscopy and electron microscopy. The results showed morphological changes that were more prominent when the truncated peptides were combined with full length *L*-PLNC8 α (Supplementary Fig. S9). A large amount of secreted or leaked material from the bacteria was observed, which formed complex thread-like networks and promoted bacterial aggregation. Furthermore, to quantify the antimicrobial effect of PLNC8 αβ, fluorescence intensity of Sytox Green (Fig. 6B), thickness of the cell wall in the TEM images (Fig. 6C) and area covered by bacteria and their leaked material in the SEM images (Fig. 6D) were determined.

### Antimicrobial activity of PLNC8 αβ in combination with antibiotics

The continuously increasing prevalence of antimicrobial resistance is a global threat to modern human medicine. A strategy that may restrict the selection and emergence of antimicrobial resistance is combination therapy. The antimicrobial effects of conventional antibiotics combined with sub-MIC concentrations of both *L*- and *D*-enantiomers of PLNC8 αβ were investigated. A final concentration of 3 µM of *L*/*D*-PLNC8 αβ was used and the MIC and MBC values were determined for *S. epidermidis* ATCC 12228 and the clinical isolate *S. epidermidis* 154. Both enantiomers of PLNC8 αβ reduced the MIC and MBC values of vancomycin, teicoplanin, rifampicin and gentamicin (Table 4). Susceptibility testing by the checkerboard microdilution method showed primarily additive effects between *L*/*D*-PLNC8 αβ and the different antibiotics. However, it was the *D*-enantiomer that showed synergistic effects with vancomycin, rifampicin and gentamicin against the clinical isolate *S. epidermidis* 154.

**Table 4:** Antimicrobial activity of PLNC8 αβ in combination with antibiotics against *Staphylococcus*.

Antimicrobial agent	*S. epidermidis* ATCC		ΣFIC	*S. epidermidis* 154		ΣFIC
	MIC	MBC		MIC	MBC	
*L*-PLNC8 αβ (µM)	6.25	12.5		6.25	12.5	
*D*-PLNC8 αβ (µM)	12.5	12.5		12.5	12.5	
Vancomycin (µg/ml)	1.5	3.1		1.5	3.1	
Vancomycin/*L*-PLNC8 αβ	0.78	1.5	0.53	<0.097	0.097	0.63
Vancomycin/*D*-PLNC8 αβ	0.78	1.5	0.51	<0.097	0.097	0.50
Teicoplanin (µg/ml)	1.5	1.5		1.5	1.5	
Teicoplanin/*L*-PLNC8 αβ	<0.097	<0.097	0.62	<0.097	<0.097	0.62
Teicoplanin/*D*-PLNC8 αβ	<0.097	<0.097	0.75	<0.097	<0.097	0.75
Rifampicin (µg/ml)	0.063	0.063		0.063	0.063	
Rifampicin/*L*-PLNC8 αβ	<0.0019	<0.0019	1.00	<0.0019	<0.0019	0.75
Rifampicin/*D*-PLNC8 αβ	0.0019	0.0019	1.00	<0.0019	<0.0019	0.50
Gentamicin (µg/ml)	0.31	0.31		>100	>100	
Gentamicin/*L*-PLNC8 αβ	0.039	0.078	1.00	25	50	0.50
Gentamicin/*D*-PLNC8 αβ	0.039	0.078	1.00	25	50	0.50

Antimicrobial activity ΣFIC between different antibiotics and *L*- or *D*-PLNC8 αβ was determined after exposure of the bacteria to a serial dilution of different antibiotic alone or in combination with 3 µM *L*/*D*-PLNC8 αβ.

The antimicrobial effect of PLNC8 αβ in combination with vancomycin, teicoplanin and rifampicin was obtained even when the final concentration of the peptides was reduced to 2 µM and 1.5 µM (data not shown). These results encouraged us to investigate the antimicrobial activity of PLNC8 αβ against *S. epidermidis* strains isolated from patients with prosthetic joint infections. The different strains were divided into two groups based on their resistance patterns against the glycopeptide antibiotics vancomycin and teicoplanin. Five strains were non-resistant and five strains were classified as heterogeneous glycopeptide intermediate *S. epidermidis* (hGISE), a group that is characterized by expressing a thick cell wall. The inhibitory activity of PLNC8 αβ was equally potent against both groups, with concentrations ranging between 6.25–12.5 µM (Table 5). However, it is obvious that the thick cell wall in hGISE renders the bacteria to be more resistant against PLNC8 αβ. The bactericidal concentrations were ≥50 µM, while the non-resistant bacteria were efficiently eliminated at concentrations ranging between 6.25–25 µM.

**Table 5:** Effects of PLNC8 αβ on heterogeneous glycopeptide intermediate strains of *S. epidermidis* (hGISE).

Strain	*L*-PLNC8αβ (µM)	
	MIC	MBC
157*	12.5	>50
126*	12.5	50
145*	6.25	>50
109*	6.25	>50
127*	12.5	50
117	6.25	25
154	6.25	12.5
138	6.25	6.25
152	6.25	12.5
124	6.25	6.25

Strains of *S. epidermidis* isolated from prosthetic joint infections, including hGISE, were exposed to *L*-PLNC8 αβ for 20 h and MIC/MBC were determined.

*Strains defined as hGISE.

Furthermore, two concentrations of *L*-PLNC8 αβ (5 and 10 µM) were used in combination with either vancomycin or teicoplanin, and the MIC, MBC and ΣFIC values were determined for MRSA and the hGISE strains 126 and 157. Interestingly, the presence of *L*-PLNC8 αβ markedly reduced the inhibitory and bactericidal concentrations of vancomycin and teicoplanin (Table 6). Similar results were achieved for all strains, except for *S. epidermidis* 157 that was unaffected. The combined effects of *L*-PLNC8 αβ and antibiotics against MRSA and hGISE 157 were additive, while the effects against hGISE 126 were synergistic.

**Table 6:** Antimicrobial effect of PLNC8 αβ with antibiotics against resistant strains of *Staphylococcus*.

Antimicrobial agent	MRSA			*S. epidermidis* 126*			*S. epidermidis* 157*		
	MIC	MBC	ΣFIC	MIC	MBC	ΣFIC	MIC	MBC	ΣFIC
*L*-PLNC8 αβ (µM)	12.5	25		12.5	50		12.5	>50	
Vancomycin (µg/ml)	1.5	3.1		3.1	3.1		6.25	6.25	
Vancomycin/*L*-PLNC8 αβ (10 µM)	<0.097	0.39		<0.097	0.78		6.25	6.25	
Vancomycin/*L*-PLNC8 αβ (5 µM)	<0.097	0.78	1.00	<0.097	0.78	0.38	6.25	6.25	1.01
Teicoplanin (µg/ml)	0.78	3.1		3.1	6.25		12.5	25	
Teicoplanin/*L*-PLNC8 αβ (10 µM)	<0.097	0.39		<0.097	0.39		12.5	25	
Teicoplanin/*L*-PLNC8 αβ (5 µM)	<0.097	0.78	0.75	<0.097	0.39	0.38	12.5	25	1.01

The bacteria were exposed to vancomycin or teicoplanin in combination with the indicated concentrations of L-PLNC8 αβ and MIC, MBC and ΣFIC were determined after 24 h.

*hGISE strains.

In addition, the truncated peptides α1–22 and β1–20 were combined together, or with either full length PLNC8 α or full length PLNC8 β, in the presence or absence of teicoplanin and rifampicin, against the clinical isolate *S. epidermidis* 154. The different peptide combinations did not inhibit bacterial growth (Supplementary Table S1). However, full length α/β1–20 and α1–22/β1–20 enhanced the effects of teicoplanin, while α1–22/full length β enhanced the effects of both teicoplanin and rifampicin.

**Figure 7:**
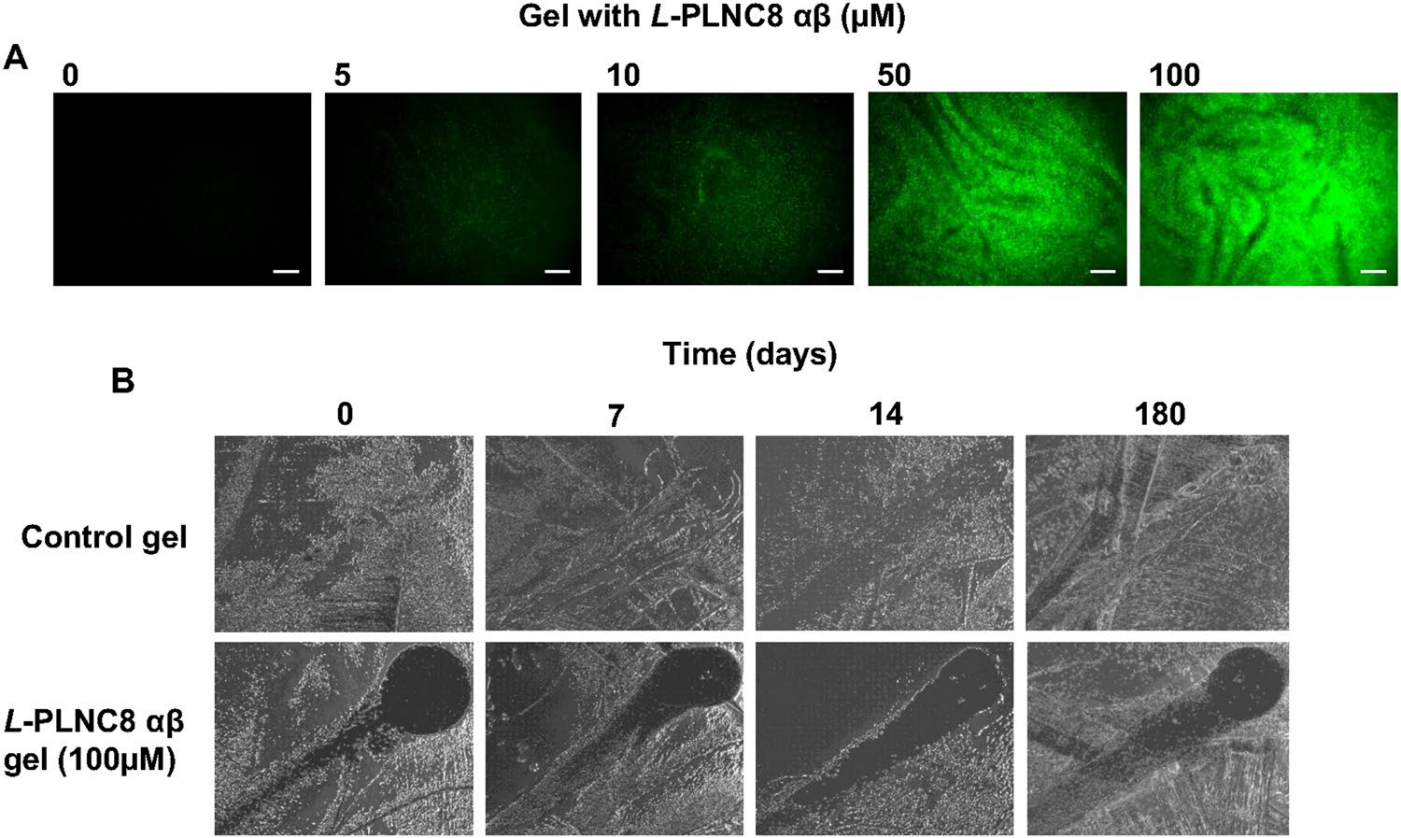
PLNC8 αβ in a formula is effective against *S. epidermidis* and retains its activity after long-term storage. (**A**) The uptake of Sytox Green by *S. epidermidis* ATCC 12228 was assayed immediately after addition of the formula, with or without PLNC8 αβ. Images were captured 1 min after treatment, scale bar is 300 µm. (**B**) The activity of this formula was further tested on blood-agar plates with *S. epidermidis* after long-term storage at 4 °C, images were captured using Olympus SZX9 at 10× magnification.

In addition, we investigated whether *L*-PLNC8 αβ exerts inhibiting effect in a gel-like formula containing glycerol and gelatine, and after long-term storage at 4 °C. Importantly, the peptides rapidly permeabilized the bacteria in a dose-dependent manner (Fig. 7A). Furthermore, the peptides in the gel retained their antimicrobial activity after long-term storage at 4 °C (>6 months), which was tested by applying the gel on a lawn of *S. epidermidis* on agar plates and incubated overnight (Fig. 7B).

## Discussion

It is evident that the spreading of antibiotic resistance is a serious global threat, putting high pressure on all sectors within healthcare systems^24^. Many species of the genus *Staphylococcus*, including *S. aureus* and *S. epidermidis*, are opportunistic pathogens and may cause severe infections in humans^25^. *S. aureus* is the most virulent species, however the low-virulent bacteria *S. epidermidis* can transform to an invasive pathogen and cause severe infections in immunocompromised patients and in association with insertion of medical devices^26^. *Staphylococcus* spp. have acquired resistance to several classes of antibiotics, such as methicillin, rifampicin, gentamycin and vancomycin^27^. Although vancomycin is an effective drug, there is an increasing development of resistance to this antibiotic^28^ and bacterial biofilm formation has been reported to decrease the activity of vancomycin^29^. Development of non-conventional therapies would be advantageous, including bacteriocins that have been suggested as a promising alternative with novel applications as next generation antibiotics^5,6^. Probiotics have previously been described for their health benefits, which are strongly considered to be associated with their production of bacteriocins that target pathogenic bacteria^5,8^. The bacteriocin Abp118 of *L. salivarius* has been shown to protect mice from infections caused by *Listeria monocytogenes*^30^, as mutation of the Abp118 gene in *L. salivarius* failed to protect the animals.

Both PLNC8 α and β are membrane active on their own, but whereas more than 5 µM of PLNC8 α was required to induce substantial perturbation of lipid bilayer integrity in a liposomal model system, less than 0.1 µM PLNC8 β caused the same effects. Since both PLNC8 α and β are cationic at physiological pH, the net charge of the lipid bilayer had a large influence on the peptide concentrations required to compromise integrity of lipid bilayers. The membrane perturbating effects were more pronounced when increasing the ratio of negatively charged lipids. Since POPS has a slightly higher negative net charge (−1) than POPG (−0.9) at pH 7.4, POPS containing lipid bilayers were more susceptible to the peptides. Including cholesterol in the lipid bilayers almost completely inhibited the effect of PLNC8. The cholesterol rich eukaryotic cell membranes are thus protected from the lytic effect of the peptides.

Due to the obvious membrane activity of PLNC8 β in bacterial lipid bilayer model systems, it could also to a certain extent permeabilize *S. epidermidis*, but this effect was not seen for PLNC8 α. However, when combining the two peptides at a 1:1 ratio, high antimicrobial activity was observed and they were extremely potent against both planktonic and surface-associated bacteria. Indeed, optimal activity of two-peptide bacteriocins, including PLNC8 αβ, is dependent on the complementary actions of two separate peptides^10^, which in turn requires GxxxG motifs and GxxxG-like motifs. PLNC8 α contains one GxxxG motif (G_11_xxxG_15_) and PLNC8 β contains two GxxxG-like motifs (S_1_xxxS_5_ and S_27_xxxG_31_). These motifs mediate close trans-membrane helix-helix interactions, leading to stabilization of the peptides, and a subsequent membrane permeabilization and bacterial cell death due to alteration in intracellular pH and electric potential^11^. The role of partition-folding coupling was supported by the induced secondary structure when PLNC8 αβ interacted with a lipid bilayer as well as the absence of antimicrobial activity when scrambling the peptide sequences.

One of the advantages of developing new antimicrobial agents based on peptides is the ability to design and chemically modify their sequences into more active or stable forms. Wang and colleagues^31^ demonstrated that peptides may be designed through analysis of the most frequently repeated amino acids residues in antimicrobial peptides of different kingdoms of life. The newly designed peptide GLK-19 was found to be more effective than human LL-37 against *Escherichia coli*. Peptide engineering has been applied on nisin Z^32^ and pediocin PA-1^33^, by replacing single amino acid residues, and successfully increased their stability and solubility. Importantly, we show that replacement of all *L*-amino acids of PLNC8 α and β with *D*-amino acids stabilized the sequences against enzymatic degradation while retaining the same antimicrobial activity as the parent peptides. These results clearly indicate that interaction of PLNC8 αβ with the bacterial membranes and the subsequent membrane disruption does not involve any specific recognition and binding to chiral components of the bacterial membrane^34,35^. The association of PLNC8 αβ to the membrane is thus likely a consequence of both electrostatic interactions between the cationic peptides and the anionic bacterial structures, such as teichoic acid and membrane lipids, and an entropy driven partitioning-folding coupling. Peptides consisting of *D*-amino acids are also considered as promising potential candidates for clinical applications, since resistance to proteolytic degradation enables use of low concentrations to achieve effective protection^36^. Furthermore, truncated versions of PLNC8 αβ showed promising effects with conserved activity, including α1–22, β7–34 and β1–20. Peptide modification through truncation is a strategy that may be applied to increase the activity of a selected peptide, increase penetration into tissues and preformed bacterial biofilms, decrease cytotoxicity against host cells and reduce the cost^37^.

Analysis of the ultrastructural changes in bacterial cells induced by PLNC8 αβ using electron microscopy revealed an irregular cytoplasmic membrane that appeared detached from the inner zone of the cell wall. The thickness of the cell wall was increased by PLNC8 αβ and leakage of a substantial amount of cell content was detected. Although pronounced bleb formation was observed following treatment with PLNC8 α, the peptidoglycan and cytoplasmic membrane were intact, indicating prevention of cytoplasm extrusion and maintenance of a normal morphological shape^38,39^. Blebbing in Gram-positive bacteria has been suggested to be formed following breakage of the tight binding between the cytoplasmic membrane and the inner wall zone^40^. Similar effects have been observed with other antimicrobials, including polymyxin B-mediated blebbing in Gram-negative bacteria^38,41,42^. The proposed mechanism has been reported to be due to an increase in outer membrane surface area, which is forced to fold outward into formation of blebs, since it is unable to expand due to the tight association with the peptidoglycan layer. Similar mechanism may explain our findings in Gram-positive bacteria involving insertion of PLNC8 α in the outer leaflet of the cytoplasmic membrane, leading to bleb formation. In contrast to PLNC8 α, extensive morphological change caused by PLNC8 β included collapsed bacterial structures with damaged cell wall and cytoplasmic membrane^43^.

Although current knowledge reports that development of resistance against antimicrobial peptides is a rare event, it is inevitable and may include proteolytic degradation, extrusion by efflux pumps and repulsion through modification of molecules in the cell envelope^17,44^. The gene *anrB*, which is a component of the ABC transporter in *Listeria monocytogenes*^45^ and the histidine kinase *cprK* (CD1352), adjacent to an ABC transporter in *Clostridium difficile*^46^ have been reported to confer increased resistance to nisin and gallidermin. Vadyvaloo and colleagues^47,48^ showed that resistant strains of *L. monocytogenes* against class IIa bacteriocins have an altered cell surface, including incorporation of *D*-alanine to teichoic acid and lipoteichoic acid that changes the cell surface to be more positively charged, and higher content of unsaturated phosphatidylglycerol, resulting in greater fluidity of the cell membrane.

It has been suggested that combination therapy inhibiting multiple cellular targets may be a successful strategy, which could potentially delay selection of resistance while reducing the dosage, and thus possible side effects^49,50^. We show that PLNC8 αβ, at sub-MIC concentrations, is able to enhance the inhibitory and bactericidal effects of conventional antibiotics. Importantly, these effects were obtained when combining PLNC8 αβ with representatives from different classes of antibiotics- cell wall synthesis inhibitors vancomycin and teicoplanin, RNA synthesis inhibitor rifampicin and protein synthesis inhibitor gentamicin. In a clinical context, a markedly lower concentration of antibiotics needed to exert bactericidal effects when combined with PLNC8 αβ would decrease the development of antibiotic resistance and reduce the risk for cytotoxic side effects. Combination therapy is a necessary and advantageous approach, with the potential to expand the limited arsenal of antibiotics against pathogens and reuse old antibiotics currently out of market. In a recent study by Pletzer and colleagues^51^, the effect of traditional antibiotics was considerably enhanced in the presence of synthetic peptides that reduced abscess size and enhanced bacterial clearance in a murine sub-cutaneous model infected with different pathogens. The suggested underlying mechanism includes increased membrane permeability and thus enhanced penetration of antibiotics. Indeed, membrane permeabilization by antimicrobial peptides may be a successful method against planktonic bacteria and biofilms, particularly their synergistic effects with conventional antibiotics when used in conjunction^52,53^.

The clinical isolates of hGISE strains are characterized by expressing a thick cell wall. Although PLNC8 αβ was equally potent at inhibiting bacterial growth, regardless of the thickness of the cell wall, it is evident that thicker cell walls makes the bacteria more tolerant against the bactericidal effects of PLNC8 αβ. Electron microscopy of *S. epidermidis* ATCC reveals that the peptides are able to affect the cell wall, however it is not clear whether this effect is direct or indirect, due to membrane permeabilization, leading to leakage and accumulation of intracellular content in the cell wall. The synergistic effect of PLNC8 αβ with glycopeptide antibiotics may be due to efficient loosening of the cell wall by PLNC8 αβ, which promotes efficient accumulation of vancomycin and teicoplanin. The difference in effects when combining PLNC8 αβ with vancomycin or teicoplanin on *S. epidermidis* 126 and 157, respectively, is probably due to their resistance patterns against glycopeptide antibiotics, where 157, but not 126, is resistant to vancomycin and teicoplanin^54^.

In this study, we show that the two-peptide bacteriocin PLNC8 αβ is potent against clinical isolates of *Staphylococcus* spp. and considerably enhances the antimicrobial activity of traditional antibiotics. These results suggest that PLNC8 αβ may be developed further to be used in clinical settings, either alone or co-administered with conventional antibiotics, against infections caused by *Staphylococcus* spp.

## Supplementary information

Dataset 1.
