# Author Correction: Plantaricin NC8 αβ exerts potent antimicrobial activity against *Staphylococcus* spp. and enhances the effects of antibiotics

**DOI:** 10.1038/s41598-020-72918-3

**Published:** 2020-09-24

**Authors:** Torbjörn Bengtsson, Robert Selegård, Amani Musa, Kjell Hultenby, Johanna Utterström, Petter Sivlér, Mårten Skog, Fariba Nayeri, Bengt Hellmark, Bo Söderquist, Daniel Aili, Hazem Khalaf

**Affiliations:** 1 Cardiovascular Research Centre, School of Medical Sciences, Örebro University, 70362 Örebro, Sweden; 2 Division of Molecular Physics, Department of Physics, Chemistry and Biology (IFM), Linköping University, 58183 Linköping, Sweden; 3 Department of Laboratory Medicine, Division of Clinical Research Centre, Karolinska Institutet, 14186 Stockholm, Sweden; 4 S2Medical AB, 58273 Linköping, Sweden; 5 Department of Infection Control, PEAS Research Institute, 58273 Linköping, Sweden; 6 Department of Clinical Microbiology, Örebro University Hospital, 70185 Örebro, Sweden

Correction to: *Scientific Reports* 10.1038/s41598-020-60570-w, published online 27 February 2020

The original version of this Article contained an error in the Abstract.

“Of the truncated peptides, β1–22, β7–34 and β1–20 retained an inhibitory activity.”

now reads:

“Of the truncated peptides, α1-22, β7–34 and β1–20 retained an inhibitory activity.”

In addition, the Authors neglected to cite a key reference related to *Lactobacillus plantarum* NC8 within the Introduction,

“Strains of *Lactobacillus plantarum* are generally recognized as probiotic and are used as dietary supplements, and have been reported to express several bacteriocins that belong to class IIb, including PLNC8 αβ^8^.”

now reads:

“Strains of *Lactobacillus plantarum* are generally recognized as probiotic and are used as dietary supplements, and have been reported to express several bacteriocins that belong to class IIb, including PLNC8 αβ^8^. These peptides have previously been sequenced and reported to be expressed in the presence of other bacteria, such as Lactococcus lactis MG 1363^9^. Full antimicrobial activity of PLNC8 is achieved through the complementary action of both PLNC8 α and β in a molar ratio of 1:16^9^.”

