# Supplementary material for: Plantaricin NC8 αβ exerts potent antimicrobial activity against Staphylococcus spp. and enhances the effects of antibiotics
Source: Sci Rep. 2020 Feb 27;10:3580. doi: 10.1038/s41598-020-60570-w (PMC7046733; doi:10.1038/s41598-020-60570-w)
Supplement: Supplementary file 1 — Dataset 1. [file 41598_2020_60570_MOESM1_ESM.pdf]

Plantaricin NC8 αβ exerts potent antimicrobial activity against *Staphylococcus* spp. and enhances the effects of antibiotics

Torbjörn Bengtsson[1#], Robert Selegård[1, 2#], Amani Musa[1], Kjell Hultenby[3], Johanna Utterström[2], Petter Sivlér[4], Mårten Skog[4], Fariba Nayeri[5], Bengt Hellmark[6], Bo Söderquist[1,6], Daniel Aili[2], Hazem Khalaf[1*]

[1] Cardiovascular Research Centre, School of Medical Sciences, Örebro University, Örebro, SE-70362, Sweden.
[2] Division of Molecular Physics, Department of Physics, Chemistry and Biology (IFM), Linköping University, Linköping, SE-58183, Sweden.
[3] Department of Laboratory Medicine, Division of Clinical Research Centre, Karolinska Institutet, Stockholm, SE-14186, Sweden.
[4] S2Medical AB, Linköping, SE-58273, Sweden.
[5] PEAS Research Institute, Department of Infection Control, Linköping, SE-58273, Sweden.
[6] Department of Clinical Microbiology, Örebro University Hospital, Örebro, SE-70185, Sweden.

[#]Authors contributed equally

[*]Corresponding author: Hazem Khalaf, hazem.khalaf@oru.se

Table of Content

Figure S1: Peptide purity 2

Figure S2: Peptide identity 3

Figure S3: Stability of *L*- and *D*-PLNC8 αβ against trypsin 4

Figure S4: Permeabilization of model lipid membranes with different lipid compositions by *L*-PLNC8 α and β 5

Figure S5: PLNC8 αβ causes rapid membrane permeabilization on liposomes 6

Figure S6: CD-spectroscopy of CD PLNC8 αβ 6

Figure S7: Haemolysis of PLNC8 αβ 7

Figure S8: Overview images of bacterial damage caused by PLNC8 α and β 8

Figure S9: Truncated peptides of *L*-PLNC8 β cause bacterial lysis 8

Table S1: Antimicrobial effect between truncated *L*-PLNC8 α and β with antibiotics against *Staphylococcus* 9

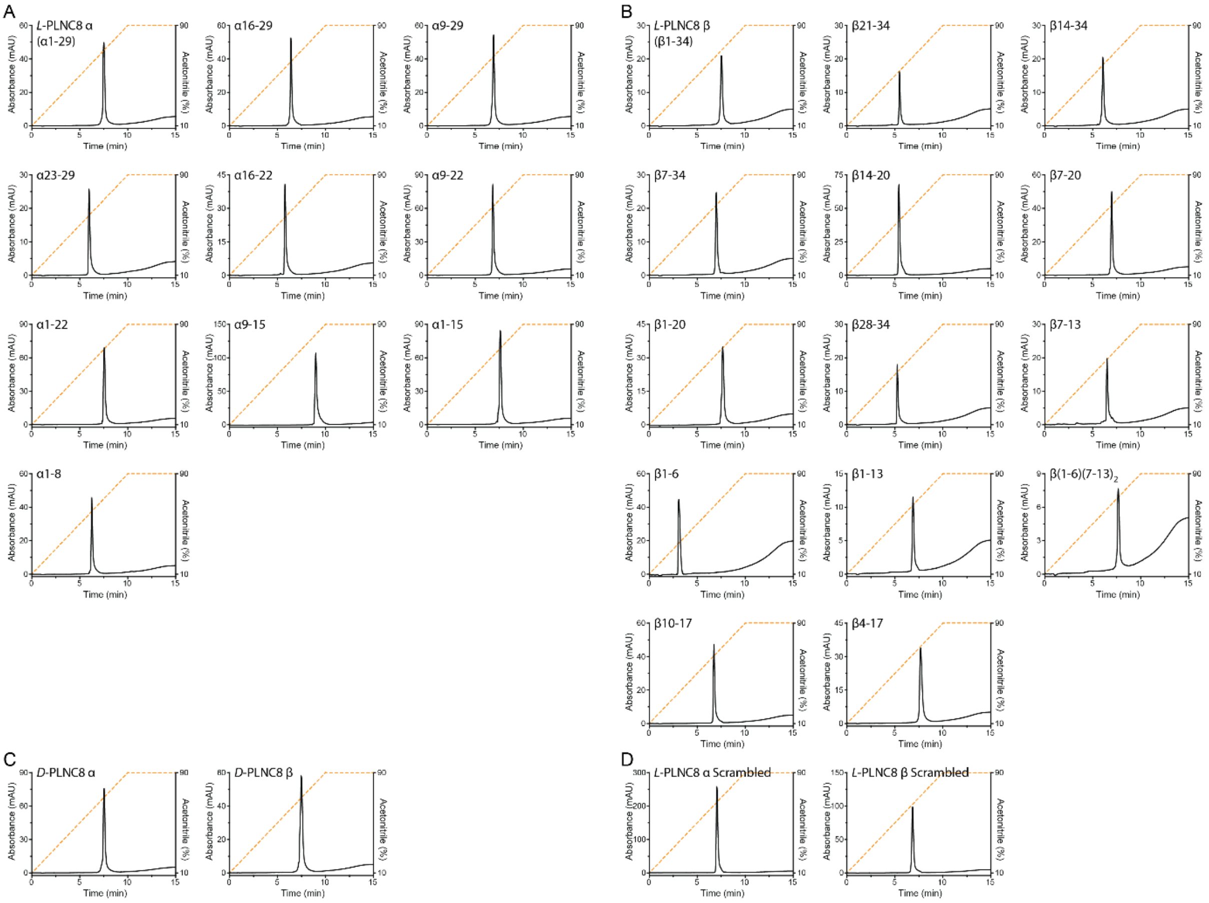

Figure S1: Peptide purity. Analytical HPLC traces of (A) native and truncated versions of *L*-PLNC8 α, (B) *L*-PLNC8 β, (C) full length *D*-PLNC8 α and β, and (D) scrambled *L*-PLNC8 α and β. Chromatograms were acquired using a C-18 analytical column (XBridge Phenyl, Waters) at a 1.5 ml/min flow rate with an aqueous gradient of acetonitrile (10-90%) containing 0.1% TFA over 10 min followed by a linear flow of 90% acetonitrile for 5 min (orange dotted line in A-D).

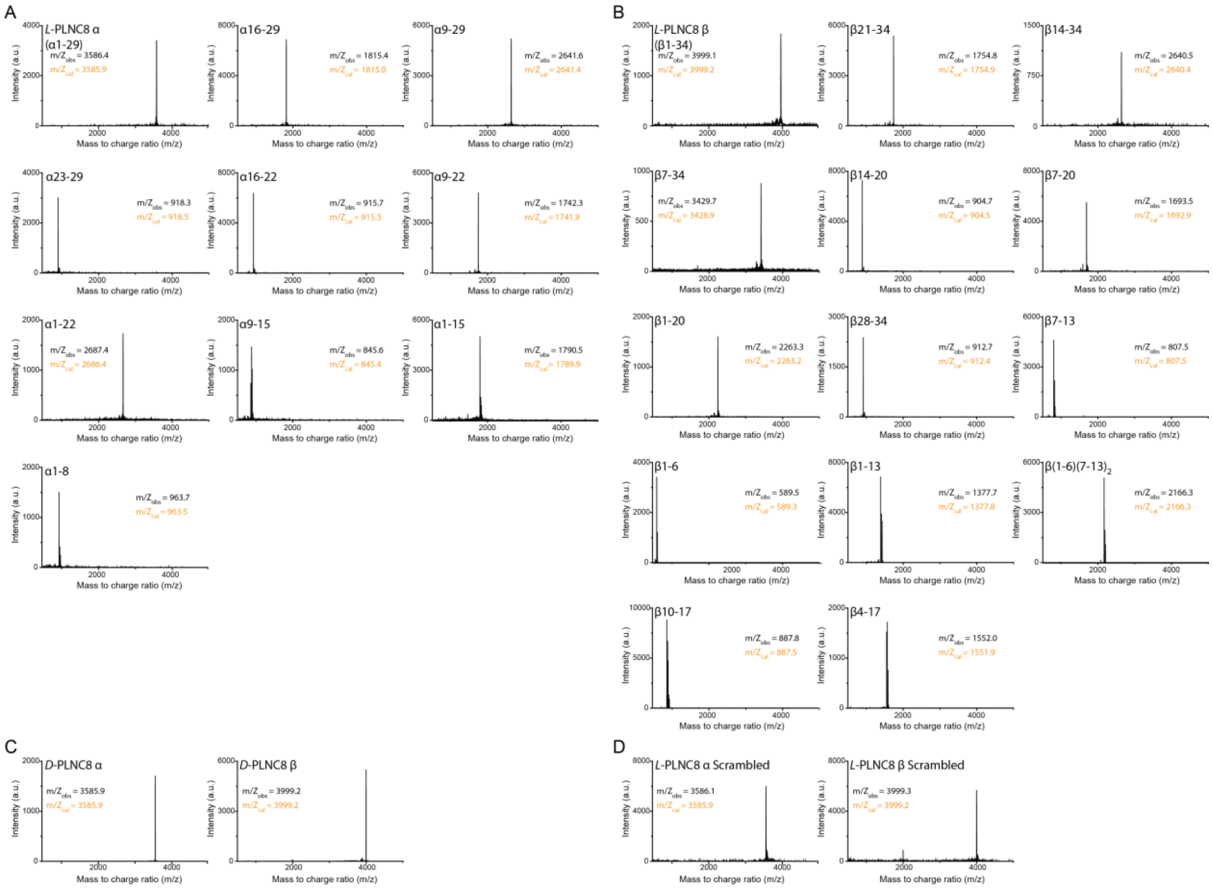

Figure S2: Peptide identity. MALDI-ToF MS of (A) native and truncated versions of *L*-PLNC8 α, (B) *L*-PLNC8 β, (C) full length *D*-PLNC8 α and β, and (D) scrambled *L*-PLNC8 α and β.

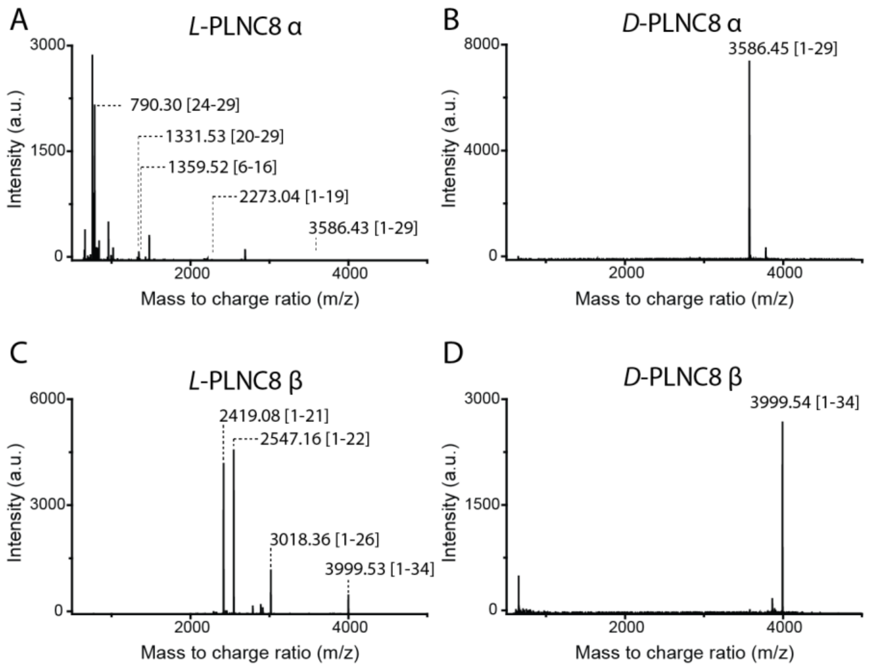

Figure S3: Stability of *L*- and *D*-PLNC8 αβ against trypsin. The peptides (100µM) (A) *L*-PLNC8 α, (B) *D*-PLNC8 α, (C) *L*-PLNC8 β and (D) *D*-PLNC8 β were treated with trypsin (5µM) in Ammonium Bicarbonate buffer (50mM) for 16 h at 37°C before being acidified (2.5% TFA), dried, suspended in H_2O + 0.1% TFA, desalted (ZipTip) and analysed by MALDI-ToF MS. Representative MS-spectra are show.

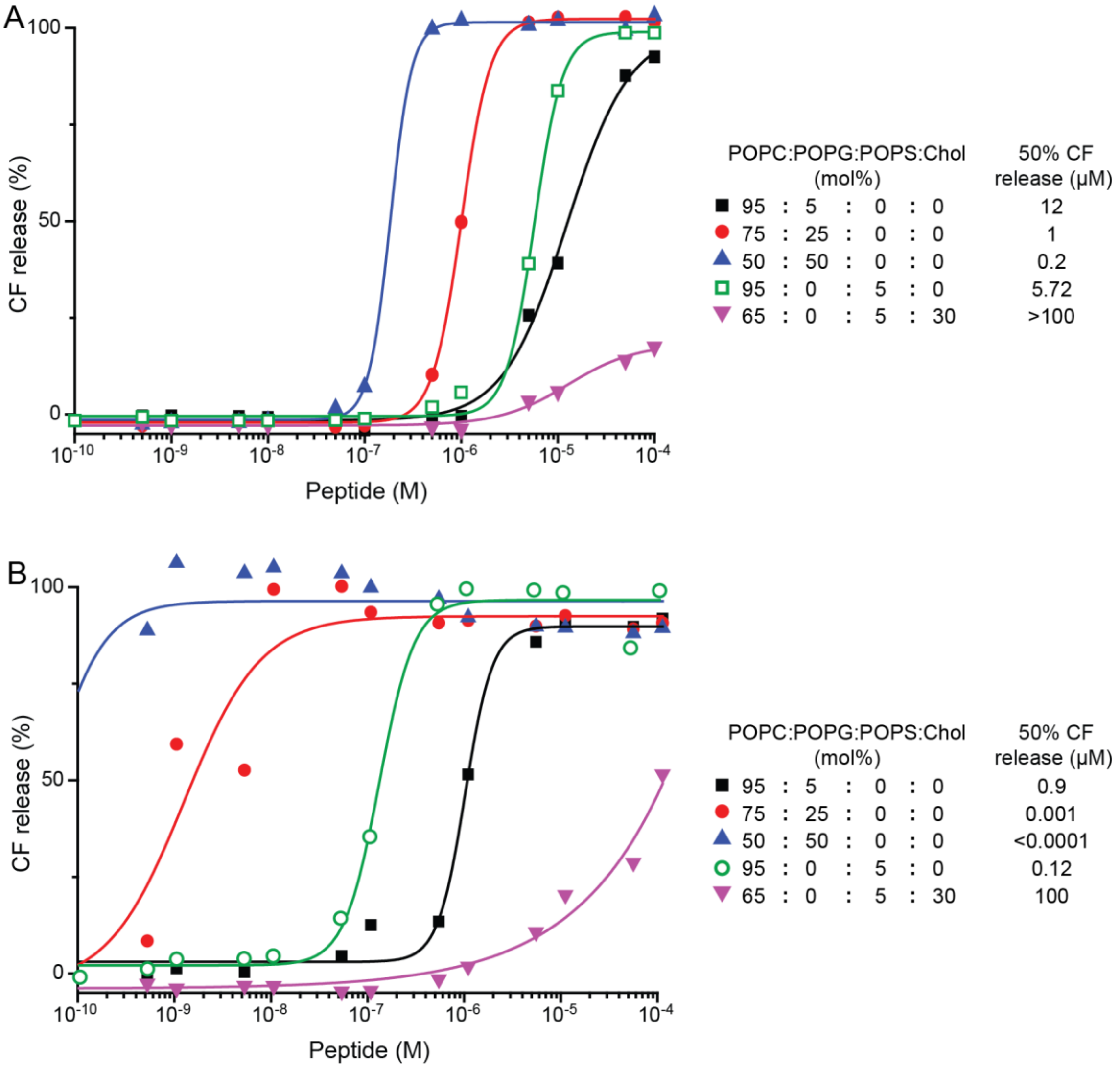

Figure S4: Permeabilization of model lipid membranes with different lipid compositions by *L*-PLNC8 α and β. CF release from liposomes exposed to *L*-PLNC8 α (A) and *L*-PLNC8 β (B) recorded after 30 min incubation. An increased ratio of negatively charge lipids (POPG and POPS) results in an enhanced permeabilization of liposomes whereas the presence of cholesterol dramatically decreases CF release.

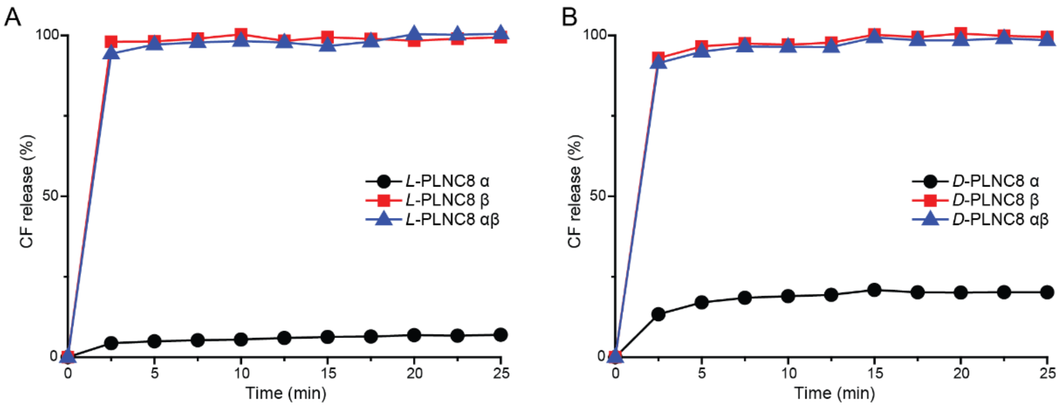

Figure S5: PLNC8 αβ causes rapid membrane permeabilization on liposomes. PLNC8 β (1 µM) and PLNC8 αβ (1:1, 1 µM), but not PLNC8 α (1 µM), of both the (A) *L*-form and (B) *D*-form, caused complete lysis liposomes (POPC:POPS (95:5)) after 2 min.

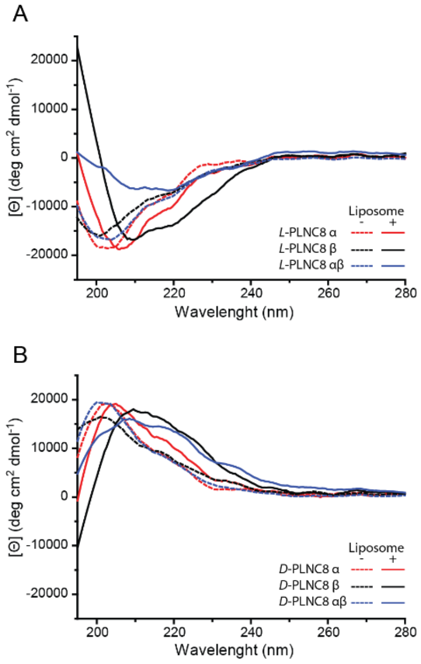

Figure S6: CD-spectroscopy of CD PLNC8 αβ. CD measurements of (A) *L*-PLNC8 αβ and (B) *D*-PLNC8 αβ (100µM each) without (dashed) and with (solid) liposomes (POPC:POPS (95:5), 0.5 mg/ml, ~660 µM) in PBS. Three repeats with PBS as background. Liposome containing samples were incubated for at least 30 min prior to measurements.

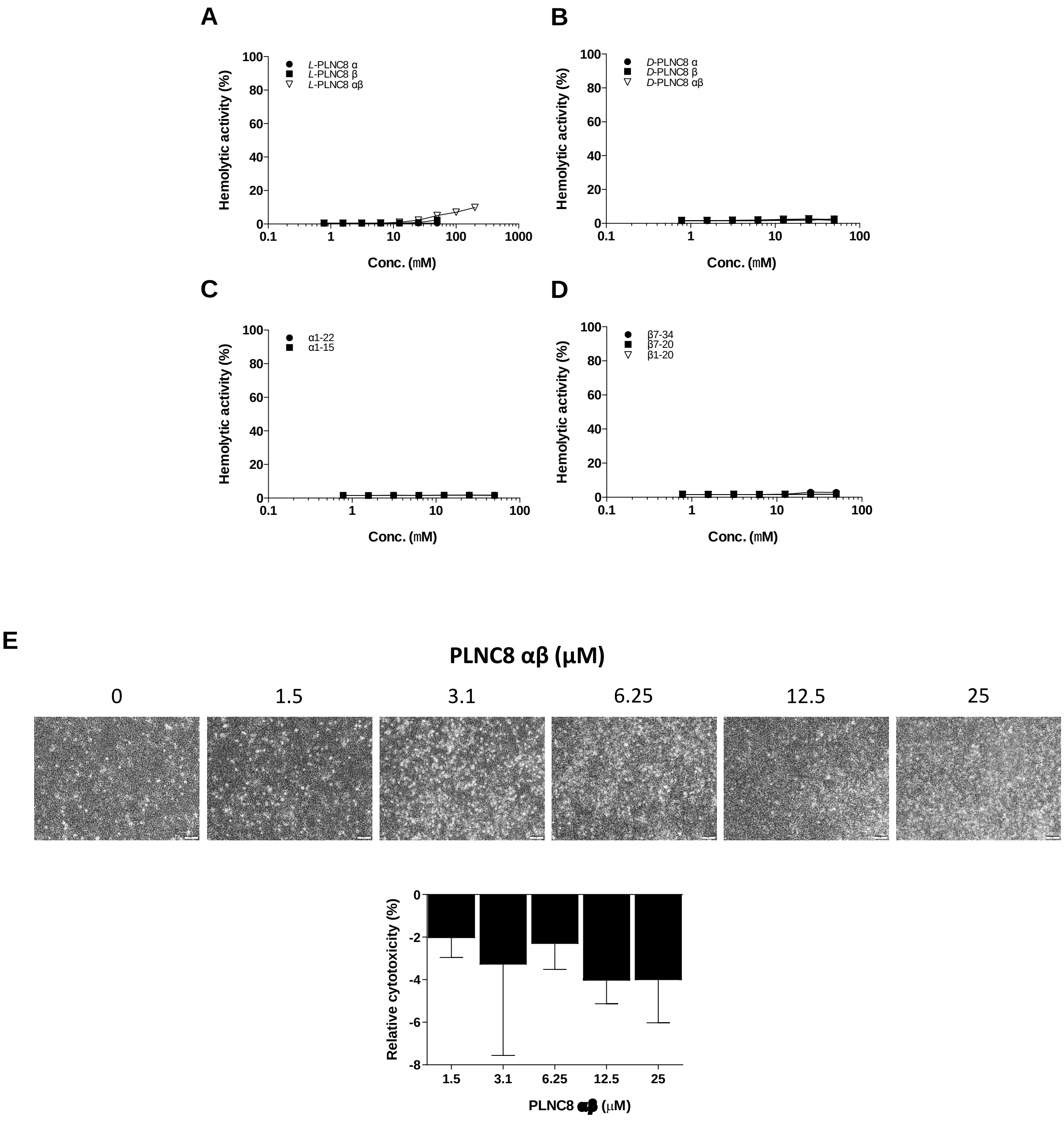

Figure S7: Haemolysis of PLNC8 αβ. Haemolytic activity of the peptides was determined on human erythrocytes with (A) *L*-PLNC8 αβ, (B) *D*-PLNC8 αβ, (C) truncated α1-15, α1-22 and (D) truncated β7-20, β1-20, β7-34, n=3. (E) Representative images and quantification, LDH activity, of the cytotoxicity of *L*-PLNC8 αβ on human keratinocytes after 24h of stimulation.

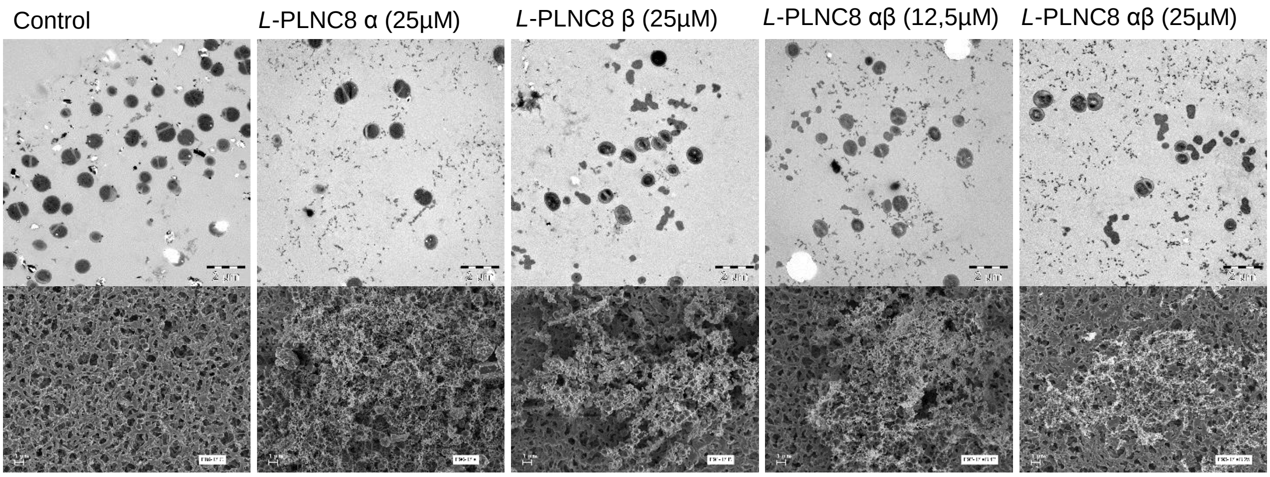

Figure S8: Overview images of bacterial damage caused by PLNC8 α and β. Representative SEM and TEM images showing *S. epidermidis* cell damage after treatment with PLNC8 α and β for 5 min.

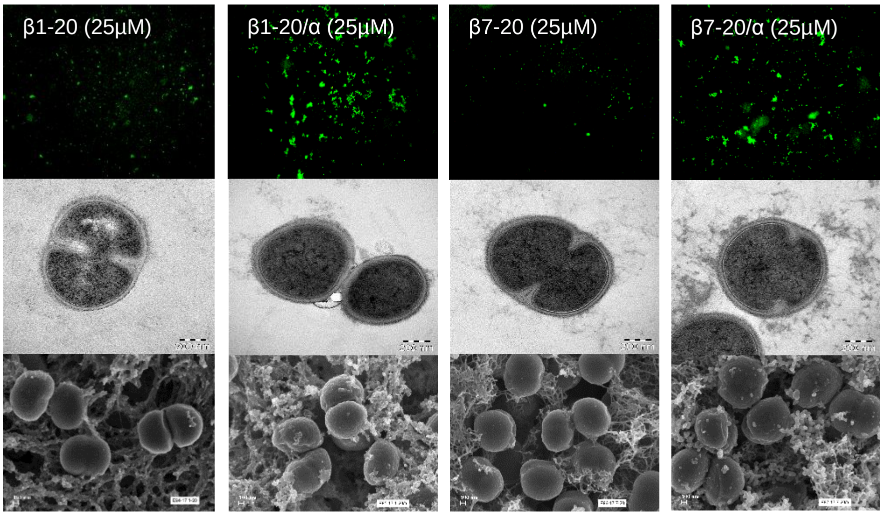

Figure S9: Truncated peptides of *L*-PLNC8 β cause bacterial lysis. The uptake of Sytox Green by *S. epidermidis* ATCC 12228, in response to 5 min treatment with different peptide combinations at a final concentration of 25 µM indicates damaged cell wall and cell membrane. Visualization of bacterial damage with scanning electron microscopy shows lysis and accumulation of a significant amount of intracellular content. The antimicrobial effect of truncated forms of *L*-PLNC8 β was significantly enhanced in combination with full length *L*-PLNC8 α.

Table S1: Antimicrobial effect between truncated *L*-PLNC8 α and β with antibiotics against *Staphylococcus*. The clinical isolate *S. epidermidis* 154 was exposed to full length α/β1-20, α1-22/full length β or α1-22/β1-20 for 20 h, with or without teicoplanin or rifampicin. The bacteria were exposed to a serial dilution of antibiotics alone or in combination with 6.25 µM of the peptides.

Antimicrobial agent	MIC	MBC
Teicoplanin (µg/ml)	1.5	1.5
Rifampicin (µg/ml)	0.025	0.05
α/β1-20 (µM)	25	>50
Teicoplanin/α/β1-20 (6.25 µM)	0.78	1.5
Rifampicin/α/β1-20 (6.25 µM)	0.025	0.05
α1-22/β (µM)	25	50
Teicoplanin/α1-22/β (6.25 µM)	<0.097	0.39
Rifampicin/α1-22/β (6.25 µM)	0.0063	0.05
α1-22/β1-20 (µM)	12.5	>50
Teicoplanin/α1-22/β1-20 (6.25 µM)	0.78	1.5
Rifampicin/α1-22/β1-20 (6.25 µM)	0.025	0.05
